# Oligomeric interface modulation causes misregulation of purine 5´-nucleotidase in relapsed leukemia

**DOI:** 10.1186/s12915-016-0313-y

**Published:** 2016-10-19

**Authors:** Aleš Hnízda, Jana Škerlová, Milan Fábry, Petr Pachl, Martina Šinalová, Lukáš Vrzal, Petr Man, Petr Novák, Pavlína Řezáčová, Václav Veverka

**Affiliations:** 1 Institute of Organic Chemistry and Biochemistry, Academy of Sciences of the Czech Republic, Flemingovo nam. 2, Prague 6, 166 10 Czech Republic; 2 Institute of Molecular Genetics, Academy of Sciences of the Czech Republic, Videnska 1083, Prague 4, 142 20 Czech Republic; 3 Institute of Microbiology, Academy of Sciences of the Czech Republic, Videnska 1083, Prague 4, 142 20 Czech Republic

**Keywords:** Nucleotidase, Cancer mutations, Relapsed ALL, Purine metabolism, Allosteric regulation

## Abstract

**Background:**

Relapsed acute lymphoblastic leukemia (ALL) is one of the main causes of mortality in childhood malignancies. Previous genetic studies demonstrated that chemoresistant ALL is driven by activating mutations in *NT5C2*, the gene encoding cytosolic 5´-nucleotidase (cN-II). However, molecular mechanisms underlying this hyperactivation are still unknown. Here, we present kinetic and structural properties of cN-II variants that represent 75 % of mutated alleles in patients who experience relapsed ALL (R367Q, R238W and L375F).

**Results:**

Enzyme kinetics measurements revealed that the mutants are consitutively active without need for allosteric activators. This shows that hyperactivity is not caused by a direct catalytic effect but rather by misregulation of cN-II. X-ray crystallography combined with mass spectrometry-based techniques demonstrated that this misregulation is driven by structural modulation of the oligomeric interface within the cN-II homotetrameric assembly. These specific conformational changes are shared between the studied variants, despite the relatively random spatial distribution of the mutations.

**Conclusions:**

These findings define a common molecular mechanism for cN-II hyperactivity, which provides a solid basis for targeted therapy of leukemia. Our study highlights the cN-II oligomerization interface as an attractive pharmacological target.

**Electronic supplementary material:**

The online version of this article (doi:10.1186/s12915-016-0313-y) contains supplementary material, which is available to authorized users.

## Background

Cytosolic purine 5´-nucleotidase (cN-II; EC 3.1.3.5) dephosphorylates purine nucleotide monophosphates with canonical activity towards inosine monophosphate (IMP) [1]. IMP represents a branch point intermediate linking major routes of purine metabolism, including de novo synthesis, the salvage pathway and oxidative degradation [2]. Therefore, cN-II serves as an essential regulatory element to maintain the balance of the nucleotide pool [3]. Unlike other nucleotidases, it is subject to complex allosteric regulation.

The activity of cN-II is regulated according to the adenylate energy charge of cells [4]. In a low energy state or anoxia, adenosine triphosphate (ATP) is depleted and free phosphate accumulates, leading to inhibition of cN-II. This hinders degradation of IMP and contributes to consequent losses of purine nucleotides via diffusion of dephosphorylated nucleosides.

The cN-II protein assembles into functional homotetramers [5], with each subunit comprising 561 amino acid residues [6]. The tetrameric assembly is formed by two identical dimers in which a dimerization site (interface A) comprises 53 intersubunit contacts. These two dimers are held together by interface B, which contains 28 interacting residues from each subunit [7].

The cN-II structure possesses the overall fold characteristic of the haloacid dehalogenase superfamily: an α/β Rossmann-like “core domain” and a smaller “cap domain.” The active site of cN-II is formed by three highly conserved regions: motif I (D52, D54, T56, and L57), motif II (T249), and motif III (K292, D351, and D359) [8]. To achieve a full catalytic activity, cN-II requires the presence of adenylate compounds, such as ATP, adenosine diphosphate or diadenosine tetraphosphate [1]. Binding of these allosteric activators induces a disorder-order transition of helix A, which spans residues 355–365. This transition places residues in the catalytic pocket into an appropriate orientation to coordinate the catalytically essential magnesium ion [9]. In addition, the cN-II sequence contains an acidic region consisting of 15 glutamates at the C terminus, which has been suggested to be involved in the protein oligomerization [10]. However, its role remains unclear due to a lack of structure-function information for this region, as X-ray crystallography studies have been feasible only with the C-terminally truncated form of cN-II (∆537–561) [7, 9].

Fluctuations in cN-II activity have been observed in various pathologies. The activity is upregulated in Lesch-Nyhan syndrome [11], an inherited disorder of purine metabolism that causes hyperuricemia and neurological symptoms. High levels of cN-II expression have also been correlated with poor prognosis in patients with hematological neoplasias and solid tumors, regardless of the therapeutic strategy [12]. In addition, cN-II activity has been implicated in decreased therapeutic efficacy of nucleoside analogs used for the treatment of cancer and viral diseases [13].

Whole-exome sequencing studies have recently revealed that somatic missense mutations in the gene encoding cN-II (*NT5C2*) are associated with relapsed acute lymphoblastic leukemia (ALL) [14, 15]. The reported mutations lead to biosynthesis of a hyperactive cN-II enzyme that boosts the viability of cancer cells in the presence of purine nucleoside analogs. These findings demonstrate that cN-II is involved in chemoresistance of leukemia cells and suggest that inhibition of hyperactive mutants might become an efficient strategy for increasing the success rates of the current chemotherapy approaches [16]. However, the molecular mechanism underlying the hyperactivity of the cN-II variants remains unknown.

In this study, we describe the biochemical and structural behavior of the three most common mutants: R367Q, R238W, and L375F. Together, these mutants represent approximately 75 % of cN-II variants observed in patients with relapsed ALL [14, 15, 17, 18]. Our work provides structural insight into the impaired allosteric regulation of ALL-specific cN-II mutants and forms a solid basis for development of novel antileukemic therapies.

## Results

### ALL-specific mutations affect regulation of cN-II

To investigate the enzymological aspects of cN-II hyperactivity, we examined kinetic properties of the most frequently occurring mutants. Kinetic parameters were determined in the presence or absence of the allosteric activator ATP. The concentration of ATP (3 mM) used in the enzyme assay reflected the physiological level of this ligand [1]. The analysis of wild-type cN-II under variable concentrations of ATP revealed that the enzyme is fully active under such conditions (Additional file 1).

Examination of the full-length wild-type cN-II revealed very low activity without ATP, as was demonstrated by the large value (≈30 mM) of the Michaelis constant (*K*_M_). Upon addition of ATP, the enzyme is stimulated, and the *K*_M_ value is decreased by 12-fold while *V*_max_ is not altered. The previous kinetic studies reported that the ATP binding affected either both *K*_M_ and *V*_max_ [1] or just *V*_max_ [19]. The divergent results may be accounted to different procedures during the protein preparation as well as to a diverse biological source of the enzyme. We used human recombinant protein expressed in bacteria, while the other studies used material isolated from human placenta or calf thymus. Nevertheless, our kinetic data are consistent with a role of cN-II activity in maintaining homeostasis of purine nucleotides [20].

Analysis of the three full-length mutant enzymes revealed that the variants exhibited different Michaelis constants but similar turnover numbers (Table 1). When compared to the wild type, all studied mutants had lower *K*_M_ in the absence of ATP, indicating that the hyperactive variants are catalytically efficient even in the absence of physiological activators. Moreover, the mutants exhibited an abnormal response to the presence of 3 mM ATP in the reaction mixture. The addition of ATP led to a large decrease in *K*_M_ (12-fold) for the wild-type enzyme, compared to only a moderate decrease (2-fold) for the R367Q. In contrast, the R238W and L375F mutants were stimulated by ATP to a higher extent than the wild-type enzyme (23-fold and 76-fold decreases in *K*_M_, respectively). Our data suggest that the R238W and L375F mutations stimulate the catalytic activity in synergy with the action of the allosteric activator, whereas the R367Q variant adopts a fully active conformation even in the absence of ATP.

**Table 1:** Kinetic parameters of cN-II enzymes in the presence and absence of ATP

Enzyme	ATP	*V*_max_ [μmol.min^-1^mg^-1^]	K_m_ [mM]	*n*	k_cat_ (s^-1^)	k_cat_/K_m_ [mM.s^-1^]
Wild-type	–	26.4 ± 4.7	33.5 ± 9.1	1.27 ± 0.38	29.5 ± 2.1	0.88 ± 0.25
Wild-type	+	31.6 ± 1.8	2.9 ± 0.8	0.90 ± 0.14	35.3 ± 2.1	12.3 ± 3.3
R367Q	–	31.4 ± 2.9	5.8 ± 1.7	0.88 ± 0.16	35.1 ± 3.3	6.0 ± 1.8
R367Q	+	28.8 ± 1.3	2.8 ± 0.8	0.85 ± 0.10	32.2 ± 1.5	11.4 ± 2.1
R238W	–	23.4 ± 1.9	12.1 ± 3.1	0.85 ± 0.09	26.1 ± 2.1	2.1 ± 0.6
R238W	+	31.2 ± 2.5	0.52 ± 0.25	0.54 ± 0.15	34.8 ± 2.8	67 ± 33
L375F	–	36.2 ± 3.9	12.2 ± 3.9	0.78 ± 0.10	40.5 ± 3.4	3.3 ± 1.1
L375F	+	29.5 ± 2.3	0.16 ± 0.08	0.49 ± 0.16	33.0 ± 2.6	206 ± 105

The *n* value refers to the calculated Hill coefficient

In summary, our comprehensive enzyme kinetics study reveals that all hyperactive variants have increased catalytic activity, even in the absence of an allosteric effector. Our results also suggest that the mutations do not have a direct catalytic effect but rather impair the allosteric regulation of the enzyme activity.

### Mutants form tetramers with altered thermostability

**Fig. 1:**
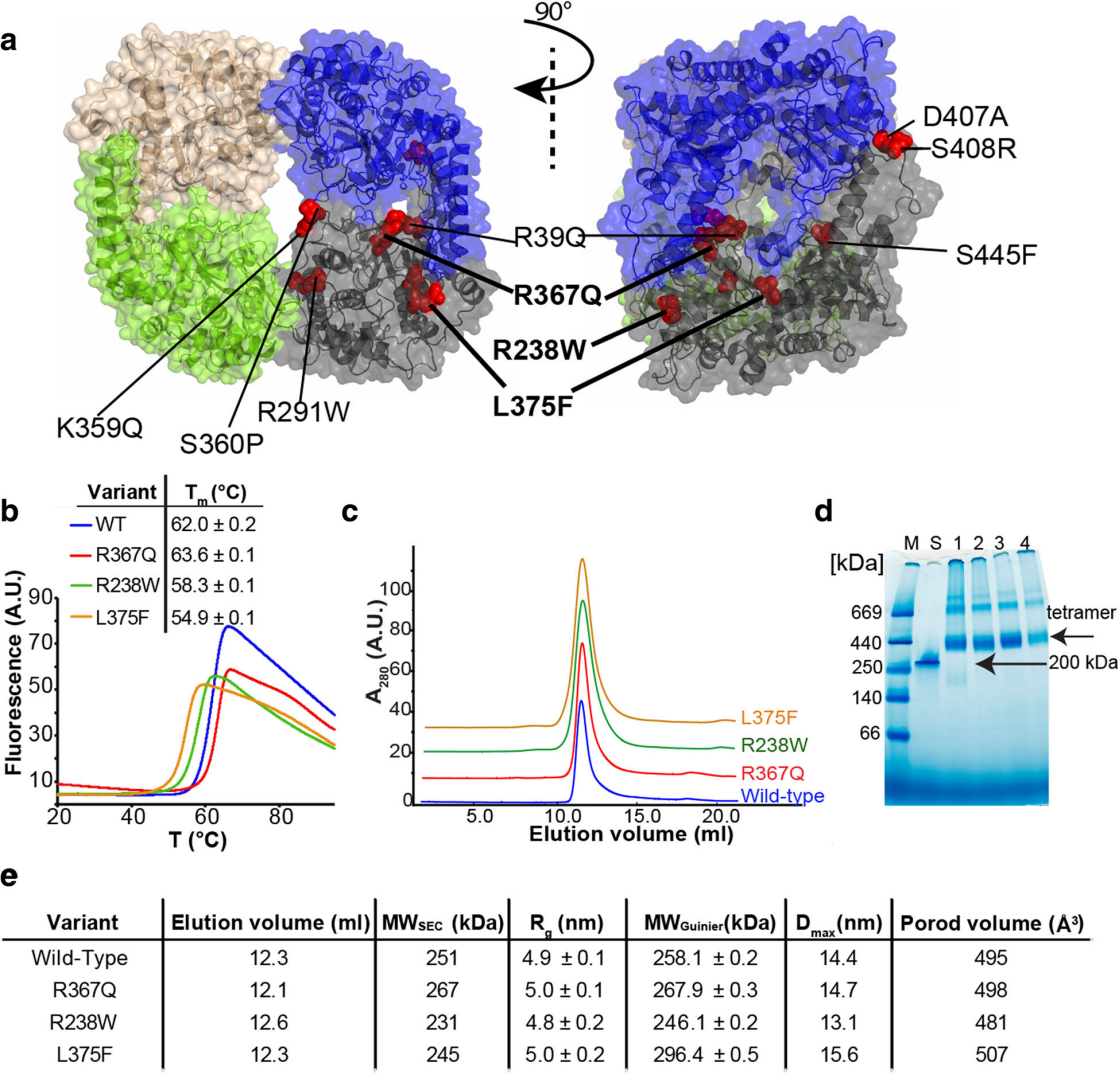
Oligomerization and thermostability of ALL-specific cN-II mutants. **a** Distribution of the identified mutations within the 3D structure of cN-II (Protein Data Bank [*PDB*] ID 2XCX; missing loops were modeled using the ModLoop server). The mutants studied in this work (R367Q, R238W and L375F) are highlighted using a *boldface font* and *thick lines*. Each subunit is represented in a different color. **b** Thermostability of cN-II proteins studied by differential scanning fluorimetry. Each sample was measured twice in two independent measurements. **c** Size exclusion chromatography of full-length cN-II protein. Elution volumes together with estimated molecular weight are summarized in the table (**e**). **d** Blue native polyacrylamide gel electrophoresis (*BN-PAGE*) of the studied full-length proteins. The protein samples (5 μg) were loaded as follows: *M* molecular weight standard, *S* β-amylase from sweet potato (200 kDa), *1* wild type, *2* R367Q, *3* R238W, *4* L375F. **e** Table summarizing data for the cN-II variants obtained from size exclusion chromatography and small-angle X-ray scattering (*SAXS*). Analysis of proteins with 1 mg/ml concentration is shown. The values of R_g_ and MW_Guinier_ were calculated by Guinier approximation; D_max_ and Porod volume were determined using fitting of distance distribution functions

As a next step, we studied the effects of the activating mutations on the cN-II conformational properties. Because these mutations are distributed close to the intersubunit contact areas in the three-dimensional (3D) structure of wild-type cN-II (Fig. 1a), we investigated whether they affect conformational stability or tetramer formation. Using differential scanning fluorimetry (DSF), we observed increased thermostability of the R367Q mutant compared to the wild type, while the R238W and L375F mutants exhibited decreased melting temperature (*T*_m_) values (Fig. 1b). To further examine the thermostability of cN-II variants, we analyzed their catalytic activity under varying temperature (Additional file 2). The wild-type protein and the R367Q mutant displayed similar behavior, exhibiting the highest activity at 50 °C. Nevertheless, the R367Q mutant had a moderately increased relative residual activity in the 45–55 °C range. In contrast, the R238W and L375F mutants exhibited maximal activity at only 45 °C, showing lower catalytic efficiency at higher temperatures. These data are consistent with the DSF observations and confirm the effect of the mutations on the conformational stability of the cN-II variants. Our results demonstrate that the mutations cause an increase (R367Q) or decrease (R238W and L375F) in the structural stability of the cN-II protein.

Next, we used DSF to monitor the thermostability of cN-II proteins under varying concentrations of ATP. Our results showed that *T*_m_ values of both the wild-type enzyme and the mutants increased in the presence of ATP by more than 5 °C (Additional file 3). Such a high degree of protein stabilization in an ATP concentration-dependent manner strongly suggests that the effects are caused by the binding event. To determine the binding affinity of the cN-II variants towards ATP, we tested the feasibility of isothermal calorimetry and microscale thermophoresis. However, we could not obtain dissociation constants due to a rather weak binding of the activating compounds to the cN-II proteins with apparent *K*_d_ in the low millimolar range [1]. As a surrogate, we used saturation transfer difference nuclear magnetic resonance (STD NMR) spectroscopy, which is a useful technique for probing of the low-affinity ligand binding [21]. We acquired the STD spectra of the variants in the presence of variable concentrations of ATP and specifically followed the enhancement in the intensity of the ATP signal. We observed a significant increase in the STD enhancement of the ATP signals for all three studied mutants relative to that of the wild-type protein at all tested ligand concentrations (Additional file 4). These experiments clearly showed that the altered allosteric regulation of the mutants is accompanied by a higher binding affinity towards the activator.

To assess the quaternary structures of the cN-II variants (full-length as well as truncated), we used size exclusion chromatography (SEC) and blue native polyacrylamide gel electrophoresis (BN-PAGE). We observed that the wild type and all the mutants migrated as biomolecules of 250–300 kDa (Fig. 1c–e), corresponding to tetrameric assemblies. This finding was further confirmed by small-angle X-ray scattering (SAXS) measurements of the cN-II protein samples at various concentrations. This analysis was feasible only for truncated enzymes due to insufficient solubility of the full-length variants. The wild-type and mutant proteins consistently exhibited radii of gyration of ~5.0 nm and molecular weights of ~260 kDa as determined by Guinier approximation, which was in good agreement with the calculation of the *P*_*(r)*_ function. In addition, similar Porod volume values of ~500 Å^3^ were obtained for all analyzed cN-II variants (Fig. 1e; representative data for the wild type are shown in Additional file 5). In summary, the results of our experiments indicate that the mutations moderately alter the protein stability but do not affect the formation of homotetramers.

### Mutation-induced structural changes are found at the oligomeric interface

To gain detailed structural insight into the misregulated cN-II mutants, we determined their 3D structures using X-ray crystallography. We analyzed C-terminally truncated variants, as previously described for wild-type cN-II [7, 9]. Our kinetic analysis, together with DSF and SEC measurements, showed that the full-length and C-terminally truncated enzymes are essentially identical (Additional files 6, 7, and 8). Therefore, the truncated variants can be considered suitable models to provide relevant structural data.

**Fig. 2:**
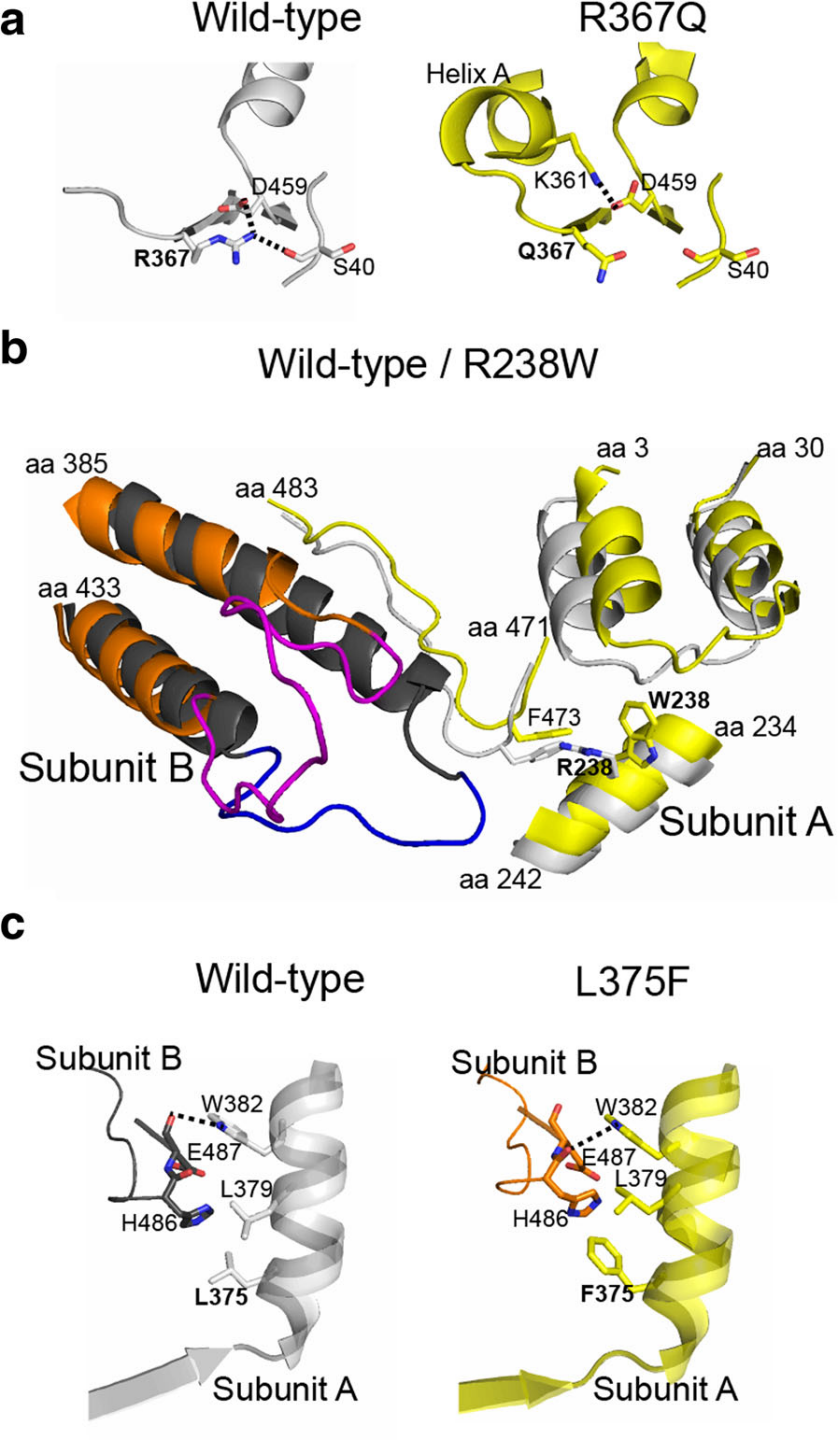
Local conformational changes in the cN-II mutants revealed by X-ray crystallography. Wild-type protein subunits are colored in *light/dark gray*, mutant subunits are colored in *yellow* or *orange*, and relevant residues are represented as *sticks*. Mutated residues are highlighted with *boldface font*. **a** The R367Q mutation disrupts the local hydrogen bonding network. **b** The R238W substitution triggers changes at the oligomeric interface. The region spanning residues 385–410 adopts a helical arrangement in the wild type, while it is unstructured in the mutant. Missing loops were modeled using the ModLoop server (highlighted in *blue* and *magenta* for wild type and mutant, respectively). **c** The L375F mutation alters local intersubunit contacts due to steric hindrance of the more bulky phenylalanine residue

Crystals of all three cN-II mutants belonged to the *I*222 space group with one molecule per asymmetric unit. We solved their structures by the difference Fourier technique using the isostructural wild-type structure [7]. Overall, the spatial arrangements of the mutants were almost identical to that of the wild type. The root-mean-square deviation (RMSD) values for superposition of 465 C_α_ atoms of individual mutants with the wild type were approximately 0.5 Å, indicating that the mutations do not induce large conformational changes in the protein. Nevertheless, detailed analysis revealed significant perturbations within the amino acid substitution microenvironment in each mutant (Fig. 2).

The R367Q mutation is located in beta-strand 14 (amino acid residues 367–371) in the proximity of helix A, which is a crucial regulatory segment of cN-II. The replacement of the positively charged arginine residue by a glutamine led to loss of residue 367 hydrogen bonding interactions with both the carbonyl group of S40 and the carboxyl group of D459. Consequently, D459 formed a compensatory polar contact with K361, a residue located in helix A, as illustrated in Fig. 2a. This interaction likely stabilizes the helix A region, which directly stimulates the catalytic activity of mutant cN-II.

The R238W mutation lies in alpha-helical region 9 (amino acid residues 231–242), which forms intersubunit contacts with a region spanning residues 385–433 of the adjacent subunit. We found structural differences between wild type and the R238W mutant mainly in residues 397–405. This segment was unstructured in the R238W mutant but adopted a helical arrangement in the wild-type protein (Fig. 2b). The helical structure was stabilized by its interactions with residues 474–480 of the adjacent subunit, and these contacts were found only in the wild-type structure. In addition, in the R238W mutant, the W238 side chain mutant was reoriented to avoid steric hindrance with F473, inducing changes within the interhelical loop at the N-terminal region (amino acid residues 3–30). Overall, the R238W mutation causes local changes, mainly at the oligomeric interface of cN-II.

The L375F mutation is located in alpha-helix 13 (amino acid residues 375–381), which interacts with a 3/10 helix (amino acid residues 485–487) in the neighboring subunit. Introduction of F375 induced a closer contact between the mutated residue and H486 of the adjacent subunit, which was accompanied by a slight displacement of the proximal L379 side chain (Fig. 2c). Consequently, this led to rearrangement of other surrounding interactions at the oligomeric interface; i.e., the NE1 atom of W382 formed a hydrogen bond with the carbonyl group of H486 in the mutant and with the D487 carbonyl in the wild type. Our data show that the L375F mutation causes local changes at intersubunit contacts.

**Fig. 3:**
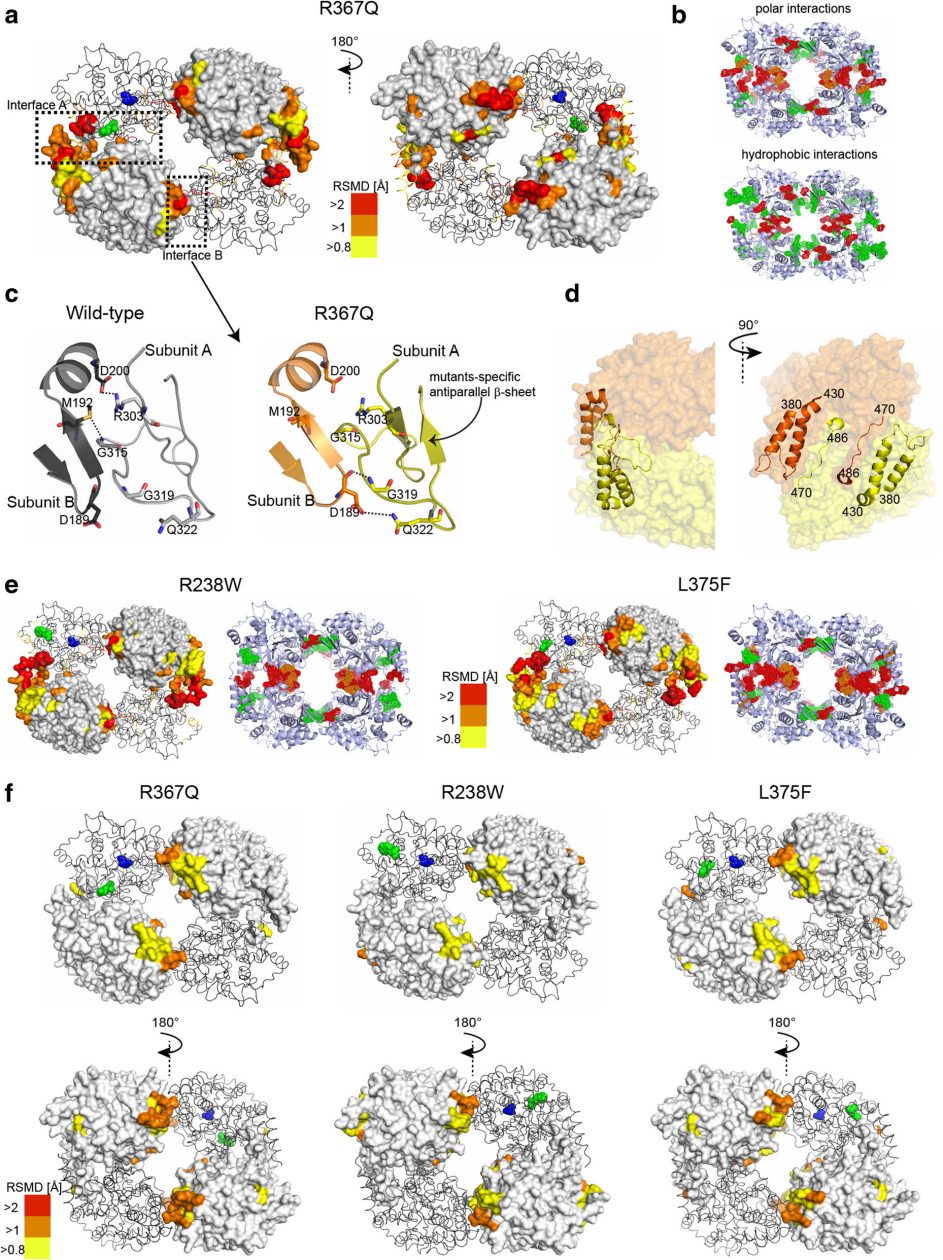
Global structural changes observed in cN-II hyperactive mutants. **a** Structural changes in the R367Q mutant revealed by its superposition with the wild-type cN-II (PDB ID 2XCX). The subunits of cN-II tetramer are represented as a *light gray surface* or *dark gray ribbons*. The residues with altered positions of C_α_ atoms are shaded according to the calculated RMSD values. The active site and mutated residues are highlighted as *blue* and *green spheres*, respectively, in one of the protein subunits. **b** Altered intersubunit interactions in the R367Q mutant. Cartoon representations (colored in *light blue*) illustrate altered intersubunit contacts in the mutant protein, i.e., loss of contact (highlighted as *green spheres*), formation of a compensatory interaction between subunits (*red spheres*), and residues with altered binding partners (*orange spheres*). **c** Detail of interface B within the wild type and the R367Q mutant. Each subunit is shown in a different color: *light* and *dark gray* (wild type) or *yellow* and *orange* (R367Q). Residues involved in altered intersubunit contacts are shown as *sticks*. The beta-sheet structure observed only in the mutants is *highlighted*. **d** Segments with the highest RMSD values located within interface A of the R367Q mutant. These regions are shown in cartoon representation, with subunits in different colors. The interhelical loop spanning residues 405–416 was not found in the crystal structure and was modeled using the ModLoop server for illustration. **e** Structural changes observed in the R238W (PDB ID 5L4Z) and L375F mutants as revealed by a superposition with the wild-type structure (surface representation) and by analysis of polar intersubunit contacts (cartoon representation). Coloring is identical as for the R367Q mutant (panels **a** and **b**). **f** Superposition of the structures obtained for the studied mutants with the ATP-bound wild-type cN-II structure (PDB ID 2XCW). The coloring is identical as for the structures in panels **a**, **b**, and **e**

To study distant structural effects of the mutations, we thoroughly analyzed superpositions of the hyperactive variants’ structures with the previously published structure of the wild-type enzyme apo form (PDB ID 2XCX) [9]. This revealed that major changes are consistently distributed across the oligomeric interface in the cN-II mutants (RMSD > 0.8 Å; Fig. 3). This observation indicates that local effects of the mutations propagate through the entire structure of cN-II and alter both contact areas responsible for oligomerization: interfaces A and B. The most significant changes at interface A (RMSD ≈ 1.2 Å) occurred within the regions between residues 385–433 and 470–486, which are in mutual contact and form a peripheral part of the oligomerization site (Fig. 3d). Major perturbations at interface B included an exclusive formation of antiparallel beta-sheets in the mutants, which very likely induced other changes in this region (RMSD ≈ 1.0 Å; Fig. 3c).

Interestingly, interface A also includes helix A (amino acid residues 355–365), which was well defined in all the mutant crystal structures. Helix A is formed only in the active state of the cN-II enzyme [9], and the stabilization of this region in the mutants is consistent with their high catalytic potency even in the absence of ATP. Furthermore, the superpositions of the mutants with the ATP-bound wild-type structure (PDB ID 2XCW) yielded reduced RMSD values in comparison to the values obtained for the wild-type structure in the absence of ATP (Fig. 3f), indicating the constitutively active state of misregulated variants.

To gain further insight into the rearrangement at the oligomeric interface of the mutants, we analyzed intersubunit contacts using Protein Interfaces, Surfaces and Assemblies (PISA) software [22]. The results confirmed differences in the pattern of intersubunit interactions at both interfaces that were accompanied by a moderate decrease in oligomerization area of the hyperactive variants (Fig. 3 and Additional file 9). In the mutants, the interface A regions were extensively rearranged and formed by unique polar interactions that were not found in the wild-type protein. These unique interactions represented nearly half of all polar intersubunit contacts in the studied cN-II variants. On the other hand, the mutants had a smaller number of hydrophobic interactions, which was reflected by a twofold lower solvation energy in interface A (Fig. 3b and Additional file 9). In contrast, the changes in interface B were located predominantly within one specific segment that was altered due to the formation of the antiparallel beta-sheet in the mutants (Fig. 3c).

In summary, the analysis of crystal structures demonstrates that all studied mutations induce topological changes almost exclusively at the oligomerization sites of the cN-II tetramer. These results show that the mutations, regardless of their position within the protein, cause essentially identical structural perturbations (Fig. 3a and e). This defines a common molecular mechanism of hyperactivation for all studied variants. X-ray crystallography revealed that the local changes induced by each point mutation propagate throughout the entire biomolecule, causing the rearrangement within the oligomeric interface of cN-II. These alterations are also accompanied by the stabilization of helix A, which had been previously identified as a major structural component in the cN-II allosteric network. Therefore, our data provide detailed mechanistic insight into how ALL-specific mutations cause hyperactivity.

### Conformational changes are accompanied by altered protein dynamics

To further examine the effects of the mutations, we studied the full-length wild-type and mutant cN-II proteins in solution. Using hydrogen/deuterium exchange (HDX) mass spectrometry, we examined the microenvironment of the backbone amide protons.

**Fig. 4:**
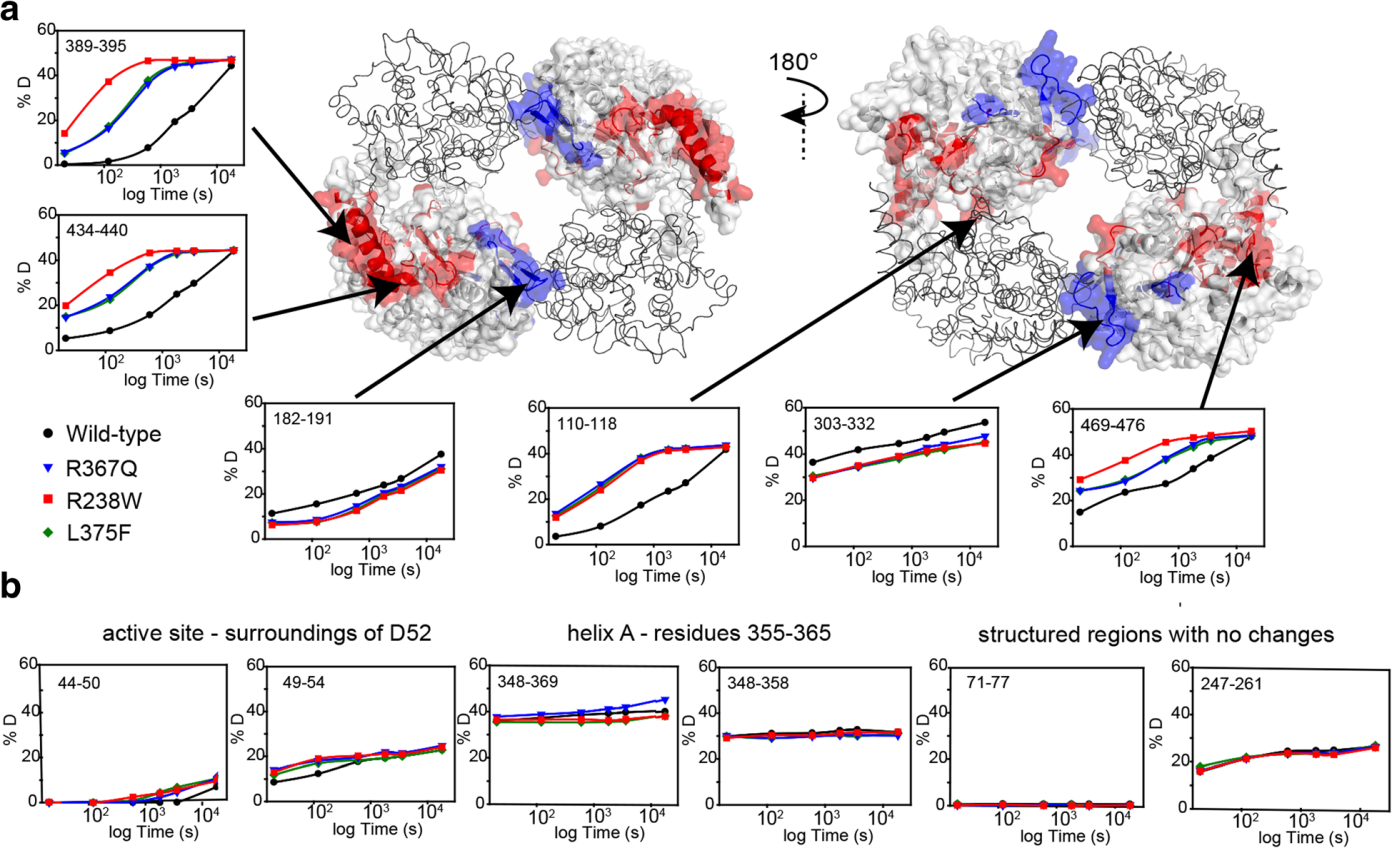
Structural changes in the cN-II mutants captured by H/D exchange. **a** Perturbations of all analyzed mutants are observed mainly at the oligomeric interface. Regions with faster (*red*) or slower (*blue*) deuteration kinetics in the mutants (related to the wild-type protein) are highlighted on the cN-II tetramer. The perturbed regions are indicated only at two subunits (surface representation) of tetramer. Graphs (percentage of deuteration) show detailed kinetics of these regions (*black circle* wild type, *blue triangle* R367Q, *red square* R238W, *green diamond* L375F). **b** HDX provides additional insight into functionally important sites in cN-II proteins. In particular, faster exchange of active site region in the mutants and high level of deuteration for helix A in all cN-II variants are shown. In addition, HDX kinetics of structured regions that do not exhibit significant changes are shown for illustration

Consistent with the crystallographic data, HDX showed that perturbations in the mutants are localized mainly at the oligomeric interface of the cN-II protein (Fig. 4). Moreover, all studied mutants exhibited altered HDX kinetics in essentially identical regions, supporting the assumption that the variants share a common structural mechanism underlying enzyme misregulation. We identified regions with altered exchange rates at both oligomeric interfaces. In particular, interface A exhibited faster HDX in the mutants than in the wild-type protein, suggesting a more open conformation. In contrast, interface B had slower exchange rates in the mutants, indicating a higher rigidity within this region when compared to the wild type. These observations are in agreement with the structural changes identified by X-ray crystallography.

Furthermore, HDX measurements provided insight into the behavior of cN-II proteins that was not captured by crystallography (Fig. 4b). Peptides spanning helix A exhibited fast HDX in the wild type as well as in the mutants, indicating a high degree of conformational flexibility for this segment (a comparison with peptides from fully structured regions of cN-II is shown in Fig. 4b). In addition, the region containing active site residue D52 (residues 47–54) exhibited increased exchange rates in each mutant. This indicates that conformational changes in hyperactive mutants are transmitted to the catalytic pocket, resulting in increased solvent accessibility at the active site. Importantly, this observation provides a direct link between the remote conformational changes and the hyperactivity of mutant enzymes.

To further analyze differences observed by HDX, we focused on the perturbed regions in crystal structures. Using RINalyzer [23], we examined residue interaction networks in the wild type and the studied mutants. This analysis revealed that most differences observed with HDX could be explained by conformational changes accompanied with altered intramolecular interactions (see comparison of HDX data with analysis of crystal structures in Additional file 10). However, a relatively small number of regions with perturbed HDX had unaltered topology in the crystal structures. This suggests that these segments had different structural flexibility that could not be captured using X-ray crystallography. Overall, HDX recorded conformational changes predominantly at the oligomeric interface of the cN-II mutants, thus supporting observations from X-ray crystallography. Moreover, HDX measurements provide complementary data about the dynamic behavior of cN-II proteins.

**Fig. 5:**
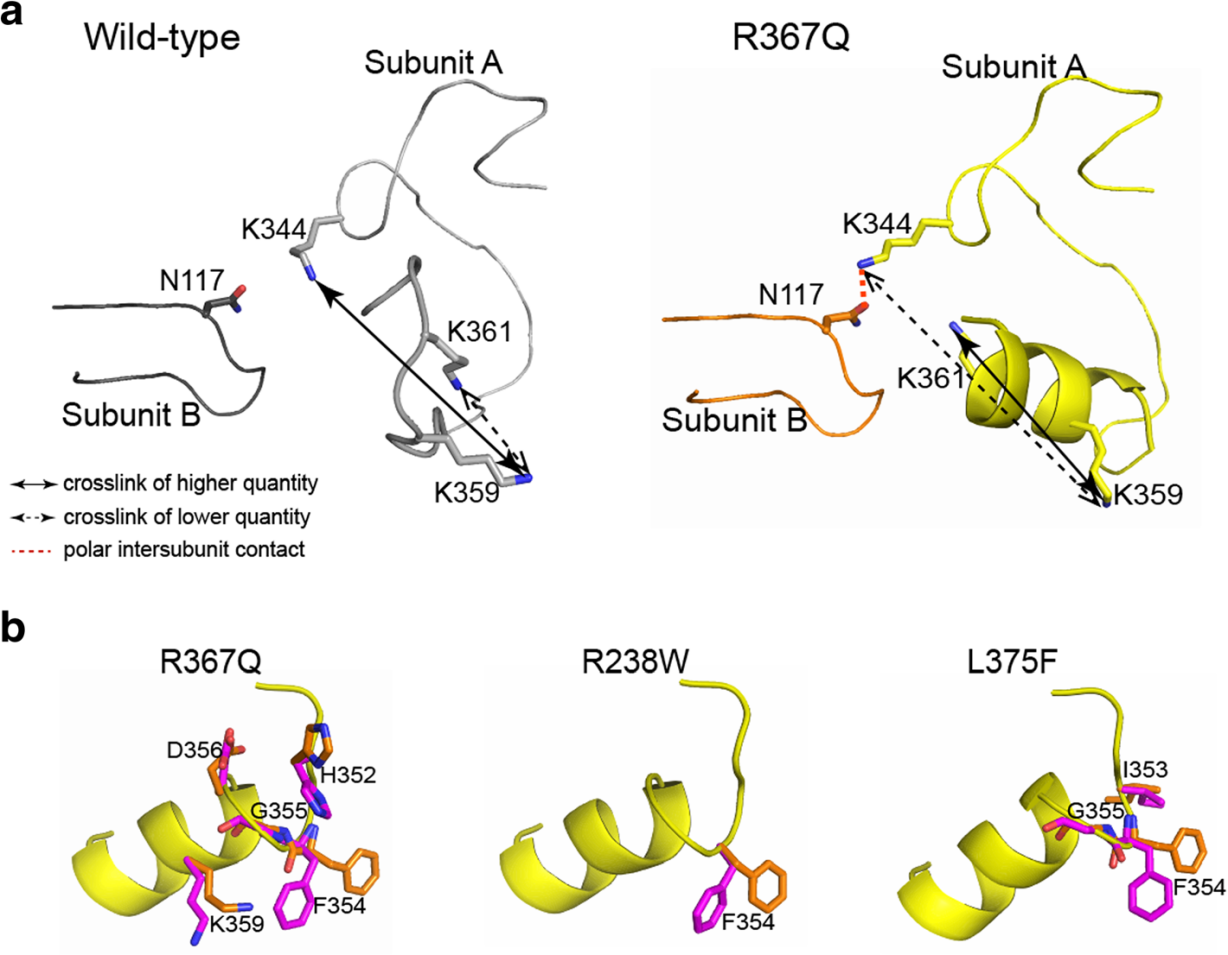
High degree of flexibility for helix A in hyperactive cN-II mutants. **a** Crosslinked lysine residues located around helix A as identified by mass spectrometry. Crosslinks K344-K359 and K359-K361 were detected even in R367Q, despite the constrained position of residues within the helical region. *Solid* or *dashed arrows* indicate lysine crosslinks with higher or lower quantity, respectively, in the particular variant. The R367Q-specific polar intersubunit contact between N117 and K344 is highlighted as a *red dashed line*. The loop spanning residues 355–365 was modeled in the wild type using the ModLoop server due to missing electron density in this region. **b** Alternative conformations of the residues distributed within or around the helix A of the cN-II mutants as observed by X-ray crystallography (highlighted as *orange* or *magenta sticks*)

To further probe the flexibility of helix A, we analyzed this segment within the wild-type enzyme and the R367Q mutant, a variant locked in a catalytically active state. Because this region is positively charged and contains three lysine residues (K359, K361, and K362), we performed amine-reactive chemical crosslinking combined with mass spectrometric analysis. Crosslinking reactions of wild-type cN-II and the mutant with bis(sulfosuccinimidyl)glutarate (BS_2_G) and bis(sulfosuccinimidyl)suberate (BS_3_) provided 13 and 9 unique crosslinked and one-site modification-containing peptides, respectively (Additional file 11). Quantitative comparison of crosslinking in the wild-type and R367Q enzymes revealed several products with altered abundance (Table 2). Interestingly, the identical crosslinking pattern and similar changes between wild type and mutant were also observed in the proteins complexed with ATP. Differentially modified sites were found in the intersubunit contact area and/or close to helix A. In particular, peptides containing a K344-K359 crosslink or one-site modification of K344 were more abundant in the wild-type cN-II. This observation is consistent with the crystal structures of cN-II proteins because K344 is localized in a region with altered intersubunit contacts (Fig. 5a). Furthermore, we detected K359-K361 crosslinks and one-site modification of K359 to a greater extent in the R367Q mutant. These changes likely reflect a lower reactivity of K344 and a subsequently lower amount of K344-K359 in the mutant.

**Table 2:** Differentially modified lysine residues revealed from mass spectrometric study of protein crosslinking in the presence and absence of ATP

			Relative abundance (wild type compared to R367Q)			
			Protein:BS_2_G molar ratio		Protein:BS_3_ molar ratio	
Peptide	Modified residues	ATP	1:20	1:50	1:20	1:50
343–361	Lys 344; Lys 359	–	77-23 ± 20	88-12 ± 12	47-53 ± 24	65-35 ± 1
		+	88-12 ± 12	89-11 ± 11	62-38 ± 33	52-48 ± 43
345–362	Lys 359; Lys 361	–	37-63 ± 4	48-52 ± 13	20-80 ± 11	19-81 ± 5
		+	55-45 ± 5	43-57 ± 23	53-47 ± 5	44-56 ± 3
345–361	Lys 359	–	24-76 ± 11	34-66 ± 15	17-83 ± 12	14-86 ± 14
		+	24-76 ± 8	32-68 ± 18	30-70 ± 8	17-83 ± 8
343–359	Lys 344	–	80-20 ± 16	82-18 ± 10	94-6 ± 4	76-24 ± 13
		+	84-16 ± 16	85-15 ± 4	100-0	93-7 ± 7

Relative abundance refers to quantitative comparison between products obtained from wild-type cN-II and the R367Q mutant. Each value represents the mean and standard deviation from three experiments

We identified a K359-K361 crosslink at the region forming helix A. However, we would expect the structurally constrained positions of lysine residues within this helical segment to prevent crosslinking (Fig. 5a). The K359-K361 product was present in the wild-type and R367Q enzymes, indicating that the helix A moves dynamically in both proteins. X-ray crystallography showed that this region is unstructured in the wild type but well-ordered in the mutant. Nevertheless, we identified several residues within helix A or in its vicinity in alternative conformations in the crystals of all mutant variants (Fig. 5b). Overall, these data show that helix A fluctuates between the ordered and disordered state, even in the active conformation of cN-II (i.e., wild type in the presence of ATP and the hyperactive variants) with a properly ordered helical segment in crystal structure. This indicates that the cN-II activity is regulated by a conformational selection or by the shifts in population of the pre-existing conformers, which are well-recognized mechanisms for protein allostery [24, 25].

In summary, mass spectrometry-based approaches provided further insight into the behavior of the studied proteins in solution. These results are consistent with those of X-ray crystallography, identifying intersubunit contact areas including helix A as key regions for regulation of cN-II activity. Mass spectrometric analyses also provided additional structural information, in particular regarding mutation-induced changes within the active site and the conformational flexibility of helix A.

## Discussion

Whole-exome sequencing studies have shown that activating mutations in *NT5C2* confer chemoresistance in ALL [14, 15]. To elucidate the molecular mechanism underlying the hyperactivity of cN-II mutants, we scrutinized the kinetic and structural behavior of three representative variants (R367Q, R238W, and L375F). We found that the ALL-specific variants are catalytically efficient even in the absence of an allosteric activator, demonstrating that the mutations cause misregulation of cN-II activity.

Using X-ray crystallography combined with HDX and protein crosslinking, we demonstrated that this misregulation is caused by conformational changes at the oligomeric interface of the protein. Our data showed that cN-II activation is associated with an altered pattern of intersubunit interactions accompanied by differences in dynamic behavior at the oligomeric interface. These changes also include stabilization of helix A, a segment that plays a crucial role in cN-II activation.

The role of intersubunit contacts in enzyme regulation was further demonstrated by targeted analysis of the available structural data (Additional file 12). In particular, the activator binding site and helix A are localized within interface A, and the access channel to the active site is formed by interface B. Moreover, superposition of the available crystal structures of wild-type cN-II (PDB IDs 2XCX and 2XCW) reveals specific changes in residues located at the oligomeric interface depending on the presence or absence of allosteric activators. In addition, the distribution of misregulation-causing mutations within the cN-II structure strongly suggests that the oligomerization sites play an essential role in allosteric regulation (Fig. 1a). A structure of wild-type cN-II in complex with noncompetitive inhibitors [26], which induce major perturbations at the regions forming intersubunit contacts, also demonstrates the importance of the oligomeric interface for cN-II regulation. Overall, we brought together several lines of evidence indicating that the oligomeric interface is involved in regulation of cN-II.

Helix A is a crucial region for cN-II allosteric regulation. Importantly, our study provided novel insight into its behavior. Structural comparison of wild-type cN-II and the hyperactive mutants revealed that helix A is well ordered only in an active conformation of cN-II. However, mass spectrometry-based techniques showed that helix A retains a certain degree of flexibility even in the active state of the enzyme. This observation was further supported by crystal structures of hyperactive mutants that revealed alternative conformations of several residues within or around helix A (Fig. 5). However, the techniques applied could not fully capture differences in the rapid order-disorder transition between the wild type and mutants. One may speculate that an active form of cN-II contains a transiently ordered helix A that is further stabilized in the crystallized proteins.

Here, we thoroughly characterized the most common ALL-specific cN-II mutants. The studied variants are representative of the structural topology of the majority of all described mutations [14, 15, 17, 18]. The mutations are localized in two regions: either close to helix A (represented by R367Q) or directly at the intersubunit contact area (represented by R238W and L375F; see Additional file 12). The positioning of the mutated residues within the protein structure corresponded to their kinetic properties and altered thermostability. In particular, the R367Q variant adopted a fully active conformation that exhibited higher thermostability than the wild-type enzyme. On the other hand, the R238W and L375F mutations stimulated catalytic activity in synergy with ATP binding and caused a decrease in protein thermostability. Based on these observations, the activating mutations can be divided into two major groups. One group comprises mutations that directly stabilize the neighborhood of helix A (R39Q, K359Q, S360P, and R367Q). This mode of action was previously suggested for K359Q, while other mutants lacked structural cues explaining hyperactivity [14]. The second group includes oligomeric interface mutations that alter intersubunit contacts (R39Q, R238W, R238G, R238L, R291W, K359Q, L375F, D407A, D407Y, S408R, P414S, and S445F). The R39Q and K359Q mutations belong to both groups, as they may stabilize helix A while affecting intersubunit contacts. These findings imply that the cN-II structure contains two hotspot regions where point mutations can cause enzyme hyperactivity.

Note that the relapsed ALL-specific *NT5C2* mutations are heterozygous and complement the wild-type allele. Consequently, cN-II tetramers can be formed by two different subunits. It is tempting to speculate that mutations induce hyperactivity even in the wild-type subunit via allosteric transmission through the oligomeric interface. Exploration of this phenomenon is beyond the scope of the present study, but should be investigated in the future.

**Fig. 6:**
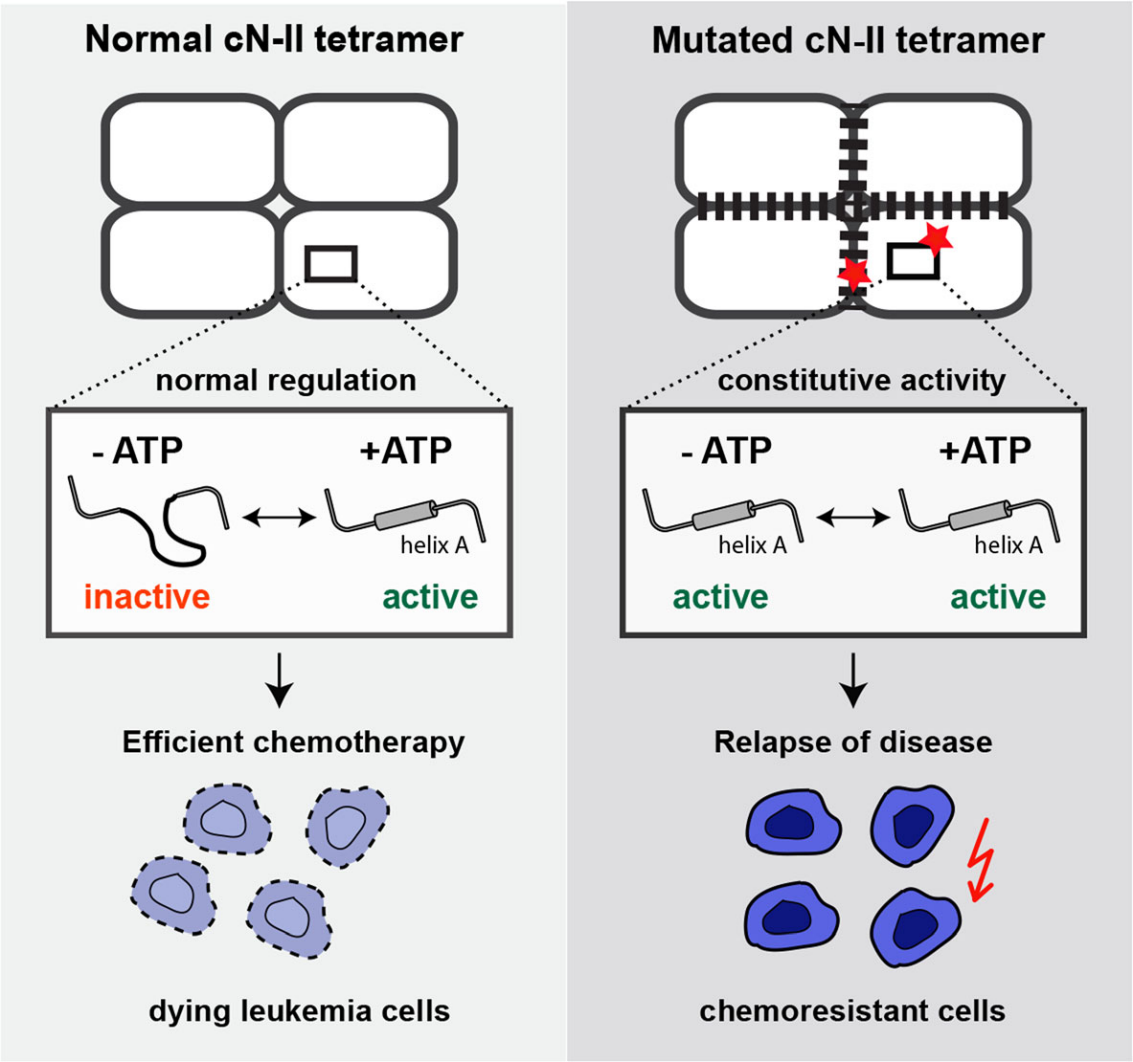
Mechanism underlying chemoresistant ALL driven by mutations in *NT5C2*. Mutations located either in the vicinity of helix A or at the oligomerization sites induce misregulation of cN-II activity through conformational modulation of the oligomeric interface, which boosts the viability of tumor cells during chemotherapy

Our enzyme kinetics analysis showed that cN-II mutants are not properly regulated and are catalytically effective even in the absence of activators. It is tempting to speculate that hyperactive mutants circumvent physiological feedback inhibition of the enzyme in order to persistently inactivate nucleoside analogs in chemoresistant leukemic cells. As a consequence, the elevated cN-II activity affects nucleotide metabolism homeostasis. We propose that misregulation of purine nucleotidase may represent a common mechanism underlying chemoresistant leukemia driven by mutations in *NT5C2* (Fig. 6).

Interestingly, misregulation of mutated PRPS1, an enzyme responsible for the first step of de novo purine synthesis, was recently confirmed as another cause for chemoresistance in relapsed ALL [27]. This strongly supports the notion that misregulation of purine metabolism affects the outcome of chemotherapy drugs targeting nucleotide metabolism. However, the list of proteins that play a role in drug metabolism remains incomplete. For example, MutT homolog 2 (MTH2) was recently implicated in the metabolism of purine nucleoside analogs [28, 29]. Indeed, the R139C variant of MTH2 causes intolerance to mercaptopurine during the treatment of ALL or Crohn’s disease [30, 31]. These findings jointly demonstrate that drug metabolism and its regulation, including the effects of genetic variations, need to be further investigated to improve the efficacy of currently used therapeutic strategies.

In summary, our data provide comprehensive insight into the allosteric regulation of cN-II, which could be used for the design of novel antileukemic drugs. Our study implies that the oligomeric interface in the cN-II protein represents a potential target for pharmacological intervention. Importantly, the intersubunit contact area contains effector sites that bind physiological ligands [7, 9, 32]. Recently, low-affinity noncompetitive inhibitors have been developed to target this site in wild-type cN-II [26]. In addition, oligomerization sites have been successfully targeted in various screening campaigns against medicinally relevant proteins (e.g., p53, tumor necrosis factor, and G-protein coupled receptors) [33]. Moreover, allosteric sites of metabolic enzymes, such as pyruvate kinase, isocitrate dehydrogenase (IDH), and glutaminase, have been proposed as important targets for specific cancer therapies [34]. Potent allosteric inhibitors of glutaminase [35] and tumor-specific IDH2 [36] mutants are currently being tested in clinical trials [37].

## Conclusion

Our study clearly demonstrates that ALL-specific mutations cause misregulation of cN-II due to allosteric modulation of the oligomeric interface, as illustrated in Fig. 6. This defines a common molecular mechanism underlying chemoresistance driven by hyperactive purine nucleotidase. Allosteric inhibition of cN-II mutants may become an effective therapeutic approach for treatment of ALL.

## Methods

### Materials

Except where stated otherwise, all compounds were purchased from Sigma-Aldrich.

### Cloning and plasmid preparation

All plasmids were derived from pET28 expressing C-terminally truncated human cN-II (Δ 536–561) with an N-terminal hexahistidine tag (Source Bioscience, UK) as previously described [7]. Site-directed mutagenesis was performed using the QuikChange Site-Directed Mutagenesis Protocol (Agilent Technologies). To prepare plasmids producing full-length proteins, the region encoding the C-terminal tail was prepared synthetically (Eurofins Scientific) and ligated into the constructs encoding the truncated variants.

### Protein expression and purification

Protein samples were prepared according to previously described procedures [7] with several modifications. Fractions collected from nickel affinity chromatography that contained cN-II protein were further purified using ion-exchange columns. Full-length proteins were dialyzed against 50 mM Bis-Tris propane (pH 7.0) containing 10 % glycerol and 2 mM tris(2-carboxyethyl)phosphine (TCEP; buffer A) and subsequently loaded onto a MonoQ column (Amersham Biosciences) and eluted with a gradient of NaCl (0–1 M) in buffer A over 30 column volumes. The C-terminally truncated variants were dialyzed against 50 mM sodium phosphate (pH 6.7) containing 10 % glycerol and 2 mM TCEP (buffer B), loaded onto a MonoS column (Amersham Biosciences), and eluted with a gradient of NaCl (0–1 M) in buffer B over 30 column volumes. Fractions containing cN-II were collected and dialyzed against 50 mM sodium phosphate (pH 7.4) containing 100 mM NaCl, 10 % glycerol, and 2 mM TCEP and stored at –80 °C.

Proteins were purified to homogeneity as judged by silver-stained SDS-PAGE. The identity of the purified cN-II proteins was validated by mass spectrometry-based peptide mass fingerprinting.

### Assay of cN-II activity

The catalytic activity of cN-II was assessed with inosine monophosphate (IMP) as a substrate according to a previously described procedure [1]. The nucleotidase activity (0.1 μM cN-II subunit) was measured in the presence or absence of 3 mM MgATP, with the IMP concentrations ranging between 10 μM to 60 mM, in 60 mM imidazole buffer (pH 7.4) containing 150 mM KCl, 1 mM MgCl_2_, and 0.5 mM dithiothreitol (DTT). The reaction was performed at 37 °C for 1–20 minutes and then chilled on ice and quenched with 0.15 M ethylenediaminetetraacetic acid. The rate of enzymatic conversion was analyzed using reversed-phase high-performance liquid chromatography. Components in the reaction mixture were separated on a Gemini 5 μm C_18_ 110 Å column (Phenomenex) by isocratic elution with 0.1 M triethylammonium bicarbonate, and signals were obtained using UV detection at λ = 262 nm. The enzyme activity was evaluated by integrating of the peaks with mobility corresponding to the standards of substrate and product. Nonlinear data fitting was performed using OriginPro 8.5 (OriginLab). The linearity of the assay was tested in the presence and absence of ATP during a reaction time of 20 minutes as illustrated in Additional file 1. Kinetic properties of the wild-type cN-II, including a response upon ATP binding, were in agreement with a previous study on human recombinant enzymes [10] and with reports deposited in the BRaunschweig ENzyme DAtabase (BRENDA).

### Differential scanning fluorimetry

Proteins (0.1 mg/ml) were dissolved in 50 mM sodium phosphate (pH 7.4) containing 100 mM NaCl, 2 mM TCEP and 8× SYPRO Orange. To analyze the effect of ATP on protein thermostability, the proteins were incubated with the ligand for 10 minutes at room temperature prior to addition of the SYPRO Orange dye. Using a LightCycler 480 (Roche) real-time detection system, the proteins were heated from 20 °C to 90 °C in increments of 0.5 °C and with 1-minute hold intervals. The signal was monitored by fluorescence detection (excitation wavelength of 470 nm and emission wavelength of 570 nm). The melting temperatures (*T*_m_) of proteins were determined as minima from first derivative curves.

### Saturation transfer difference NMR

All data for cN-II proteins were collected at 25 °C using a Bruker Avance III™ HD 850 MHz spectrometer equipped with a 5 mm CPTCI ^1^H/^13^C/^15^N/D Z-GRD cryoprobe. In order to compare the relative ATP affinities towards the cN-II variants, ^1^H STD NMR spectra were acquired using 350-μl samples of 1.5 μM cN-II proteins dissolved in 50 mM sodium phosphate buffer (pH 7.4) containing 100 mM NaCl, 2 mM TCEP, and 5 % D_2_O/95 % H_2_O. The frequency used for non-selective irradiation of the protein signals was 666 Hz (0.78 ppm), using a 50-ms shaped pulse Eburp2.1000 at a power of 40 dB. The spectra were acquired with a free induction decay (FID) resolution of 0.83 Hz, and the typical experimental time was 20 minutes.

### Size exclusion chromatography

Proteins were dissolved in 50 mM sodium phosphate (pH 7.4) containing 100 mM NaCl and 2 mM TCEP and analyzed using a Superdex 200 10/300 GL column. The column was calibrated with a high molecular weight protein standards kit (Amersham Biosciences). For analysis, 200 μl of cN-II protein samples (0.5 mg/ml) were injected.

### Small-angle X-ray scattering

Truncated proteins of varying concentrations (from 0.25 to 2 mg/ml) dissolved in 50 mM sodium phosphate (pH 7.4) containing 100 mM NaCl, 2 mM TCEP, and 5 % glycerol were analyzed at the BM29 beamline at the European Synchrotron Radiation Facility (ESRF) (Grenoble) with a Pilatus 1 M detector. The measurements were performed at a sample-detector distance of 2.864 m and a wavelength of 0.992 Å. Data were processed with PRIMUS [38], and the scattering curves were analyzed using Guinier approximation to calculate the forward scattering *I*_*(0)*_ and radius of gyration (*R*_g_). A solution of β-amylase from sweet potato (200 kDa) was used as a reference sample. Distance distribution functions *P*_*(r)*_, maximum particle dimensions *D*_max_ [39], and Porod volume were computed with GNOM [40]. All used programs are included in the ATSAS software package [41].

### Blue native polyacrylamide gel electrophoresis (BN-PAGE)

BN-PAGE was performed using 4–16 % polyacrylamide Bis-Tris precast gels (Invitrogen) at room temperature according to the manufacturer’s protocol with high molecular weight native marker kit (Amersham Biosciences) and β-amylase from sweet potato as protein standards. A 5-μg aliquot of protein was loaded in each lane as described previously [42].

### X-ray crystallography

The C-terminally truncated mutant proteins lacking residues 537–561 were crystallized as described for wild-type cN-II [7]. The proteins (8 mg/ml) in 100 mM sodium citrate (pH 6.0) containing 2 mM TCEP were crystallized using the hanging drop vapor diffusion technique. The precipitant for each mutant was as follows: R367Q, 100 mM MES/imidazole (pH 6.5) containing 200 mM NaCl, 30 % glycerol, and 10 % PEG 4 k; R238W, 100 mM MOPS/HEPES-Na (pH 7.5) containing 100 mM NaCl, 15 % glycerol, and 10 % PEG 4 k; L375F, 200 mM MES/imidazol (pH 6.5) containing 100 mM NaCl, 30 % glycerol, and 10 % PEG 4 k. The crystals grew in 1.5-μl drops of 2:1 protein:precipitant solution for 1 week at room temperature and subsequently were transferred into a drop of perfluoropolyether for 20 s and then flash-frozen in liquid nitrogen. Diffraction data were collected on BL14.1 operated by the Joint Berlin MX Laboratory at the BESSY II electron storage ring (Berlin-Adlershof, Germany) [43]. Data processing was performed using the XDS software package [44]. The 3D structure of the mutants was solved by molecular replacement with the structure of wild-type enzyme (PDB ID 2J2C) as a template using the MOLREP program [45]. Refinement was performed with REFMAC 11.0 [46], including translation/libration/screwing (TLS) refinement [47] with 12 TLS groups at a later stage of refinement. Manual rebuilding of the model was done using Coot [48]. The MolProbity server [49] was used for assessment of the final model quality. Structural statistics are shown in Additional file 13. The protein structures were analyzed using software included in the CCP4 package [50], namely PISA [22], LSQKAB [51], and BAVERAGE. In addition, the residue interaction network was assessed with RINalyzer [23]. Structural models were visualized using PyMOL software (DeLano Scientific). Missing loops were modeled using the ModLoop server [52] to illustrate the possible position of these regions in the protein structure. These are not part of the models deposited in the PDB under accession codes: 5K7Y (R367Q), 5L4Z (R238W), and 5L50 (L375F).

### Hydrogen/deuterium exchange mass spectrometry

Full-length proteins (1 mg/ml) were dissolved in 20 mM sodium carbonate (pH 7.4) containing 100 mM potassium chloride, 1 mM magnesium chloride, and 0.5 mM TCEP. Proteins were diluted to a final concentration of 0.2 mg/ml with the same buffer prepared in D_2_O (pD 7.4) to initiate HDX that was subsequently quenched at varying time points by adding the same volume of 0.25 M glycine-HCl buffer (pH 2.3) and immediately freezing the samples on liquid nitrogen. Liquid chromatography-mass spectrometry (LC-MS) analysis, including on-line pepsin digestion, data acquisition, processing, and evaluation, was performed according to a previously described procedure [53].

### Chemical crosslinking studied by mass spectrometry

Full-length proteins (0.2 mg/ml) dissolved in 50 mM triethylammonium bicarbonate (pH 7.4) containing 50 mM NaCl, 1 mM MgCl_2_, and 0.5 mM DTT were incubated with the crosslinking agents BS_2_G and BS_3_ in protein:crosslinker ratios of 1:20 and 1:50, respectively. Reactions were carried out at room temperature for 1 h and then quenched with 0.15 mM ethanolamine. Crosslinked proteins were reduced with DTT, alkylated with iodoacetamide, and digested using trypsin. To compare the crosslinking pattern of wild type and R367Q, isotopically labeled crosslinkers were used, and a quantitative comparison of light/heavy crosslinked peptides was performed by LC-MS as described elsewhere [54].

Crosslinked proteins were analyzed using SDS-PAGE to check that the reactions did not yield nonspecific aggregates (Additional file 14).

## Additional files

Additional file 1: Validation of the enzyme assay. **a** The cN-II activity under varying concentrations of ATP. The analysis was performed using 1 mM IMP as substrate. The points represent mean values with standard deviation from four measurements. **b** The test of linearity for catalyzed reaction for cN-II in the presence and absence of ATP. The analysis was performed with 5 mM IMP as substrate. (TIF 421 kb)
Additional file 2: Temperature dependence of cN-II catalytic activity. The activity values are relative to the values obtained at 40 °C with 1 mM IMP as substrate. The points represent mean values from two independent measurements. (TIF 452 kb)
Additional file 3: Thermostability of cN-II proteins in the presence of ATP studied by differential scanning fluorimetry. **a** Representative curves of the wild-type and mutant proteins in the presence or absence of 3 mM ATP. **b** The *T*_m_ values of the cN-II proteins under varying concentrations of ATP. Each point represents the mean value from two measurements. (TIF 1192 kb)
Additional file 4:
^1^H STD NMR spectra of ATP at various concentrations in the presence of cN-II variants. The ATP reference ^1^H NMR spectrum (standard atom numbering) is shown at the *bottom*. (TIF 1286 kb)
Additional file 5: Representative data from SAXS measurements: analysis of wild-type cN-II (1 mg/ml). **a** Processed scattering curve. **b** Guinier analysis. **c** Distance distribution analysis including calculation of the *P*_*(r)*_ function. (TIF 836 kb)
Additional file 6: Kinetic parameters of the C-terminally truncated enzymes in the presence and absence of ATP. The *n* value refers to the calculated Hill coefficient. (DOCX 15 kb)
Additional file 7: Differential scanning fluorimetry of the C-terminally truncated cN-II variants in the presence or absence of 3 mM ATP. Truncated proteins are stabilized upon ATP binding, as described for full-length variants. Mean values and standard deviations from duplicates of two independent measurements are listed. (DOCX 14 kb)
Additional file 8: Size exclusion chromatography of the C-terminally truncated cN-II proteins that were studied by X-ray crystallography. Each variant formed tetramers as reported for full-length proteins. The column was calibrated using a high molecular weight protein standard kit (Amersham Biosciences). (TIF 284 kb)
Additional file 9: Oligomeric interface of cN-II crystals analyzed by PISA. N_HB_ and N_SB_ represent the number of identified hydrogen bonds and salt bridges, respectively. ∆^i^ *G* is the energy of solvation. (DOCX 15 kb)
Additional file 10: Regions with perturbed deuteration kinetics analyzed in crystal structures using RINalyzer. The ‘+’ and ‘-’ symbols refer to the presence or absence of structural changes, respectively. (DOCX 15 kb)
Additional file 11: Cross-linking of the wild-type enzyme and R367Q mutant: modified residues identified and quantified using mass spectrometry. Relative abundance refers to a quantitative comparison between products obtained from wild-type and R367Q. The type of crosslinking (i.e., intermolecular, intramolecular or hanging) was inferred from the mass spectrometric data or from the crystal structure. Each number represents the mean value and standard deviation from three experiments. (DOCX 18 kb)
Additional file 12: Structural analysis demonstrates an important role for the oligomeric interface in cN-II allosteric regulation. **a** Position of helix A (*red dots*) and the ATP binding site (*orange dots*) within the cN-II tetramer. **b** The access channel towards the active site is formed by interface B. Each subunit is highlighted in a different color; the active site residue D52 is shown in *red*. **c** Superposition of wild type in its free state and in complex with ATP (PDB IDs 2XCX and 2XCW). Differences in positions of C_α_ atoms are highlighted in the structure of the apo form (PDB ID 2XCX). The active site residue (D52) is shown as *blue spheres*. **d** Location of ALL-specific mutations reveals two hotspot regions. Each subunit is depicted in a different color, and helix A is shown as an *orange cylinder*. Mutated residues are highlighted as *red spheres* within one subunit shown in ribbon representation (*wheat colored*). (TIF 10513 kb)
Additional file 13: Crystal parameters, data collection and refinement statistics. (DOCX 20 kb)
Additional file 14: Representative SDS-PAGE of the wild-type cN-II protein upon crosslinking reaction with bis(sulfosuccinimidyl)glutarate (BS_2_G) and bis(sulfo-succinimidyl)suberate (BS_3_). *M* and *N* refer to molecular weight marker and non-modified sample, respectively. The protein:crosslinker molar ratio is indicated at the top of each lane. The arrows indicate the number of subunits contributing to crosslinking products. (TIF 1253 kb)
