# Supplementary figures and images for: Oligomeric interface modulation causes misregulation of purine 5´-nucleotidase in relapsed leukemia
Source: BMC Biol. 2016 Oct 19;14:91. doi: 10.1186/s12915-016-0313-y (PMC5070119; doi:10.1186/s12915-016-0313-y)

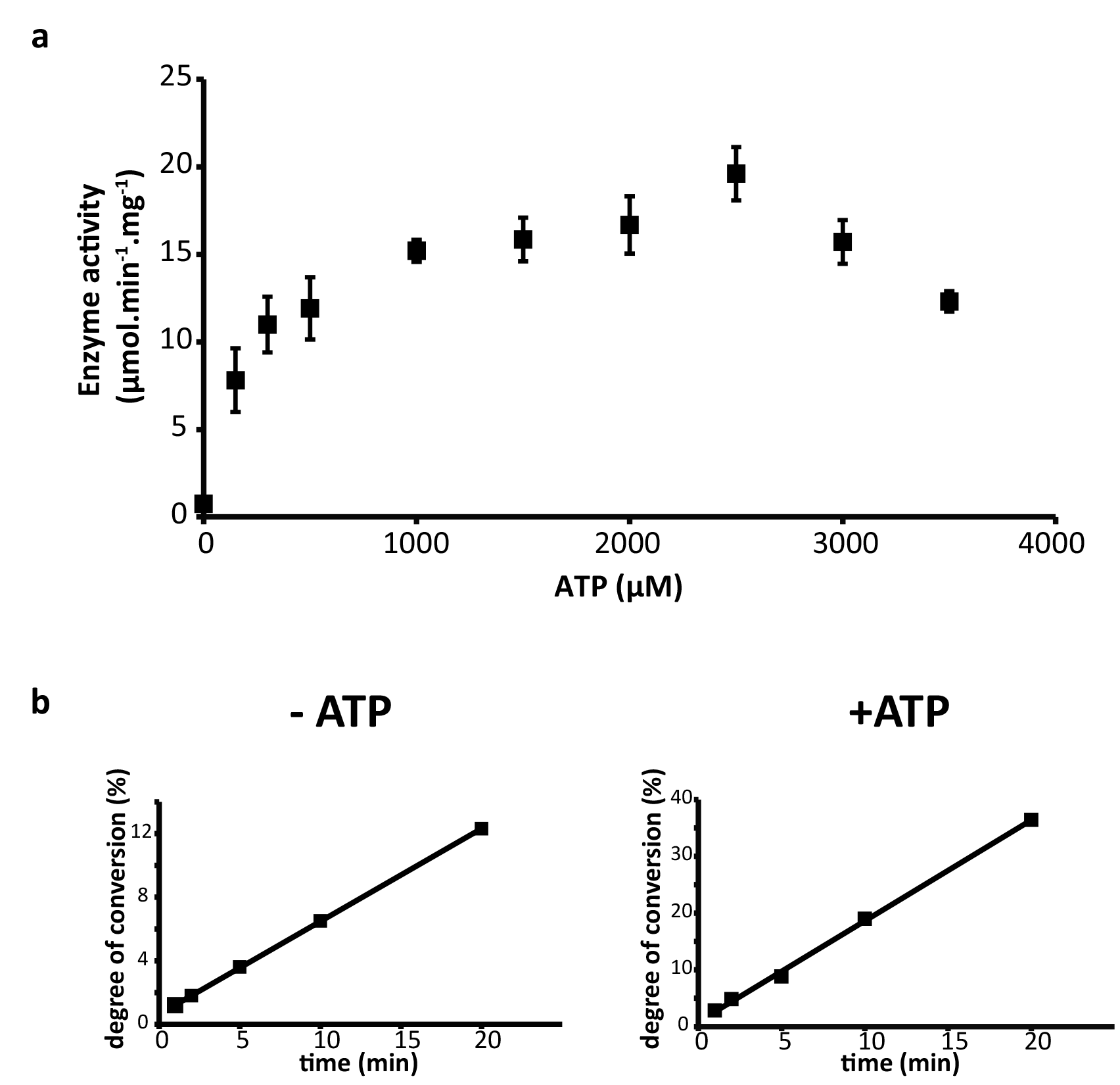

Supplement: Additional file 1: — Validation of the enzyme assay. a The cN-II activity under varying concentrations of ATP. The analysis was performed using 1 mM IMP as substrate. The points represent mean values with standard deviation from four measurements. b The test of linearity for catalyzed reaction for cN-II in the presence and absence of ATP. The analysis was performed with 5 mM IMP as substrate. (TIF 421 kb) [file 12915_2016_313_MOESM1_ESM.tif]

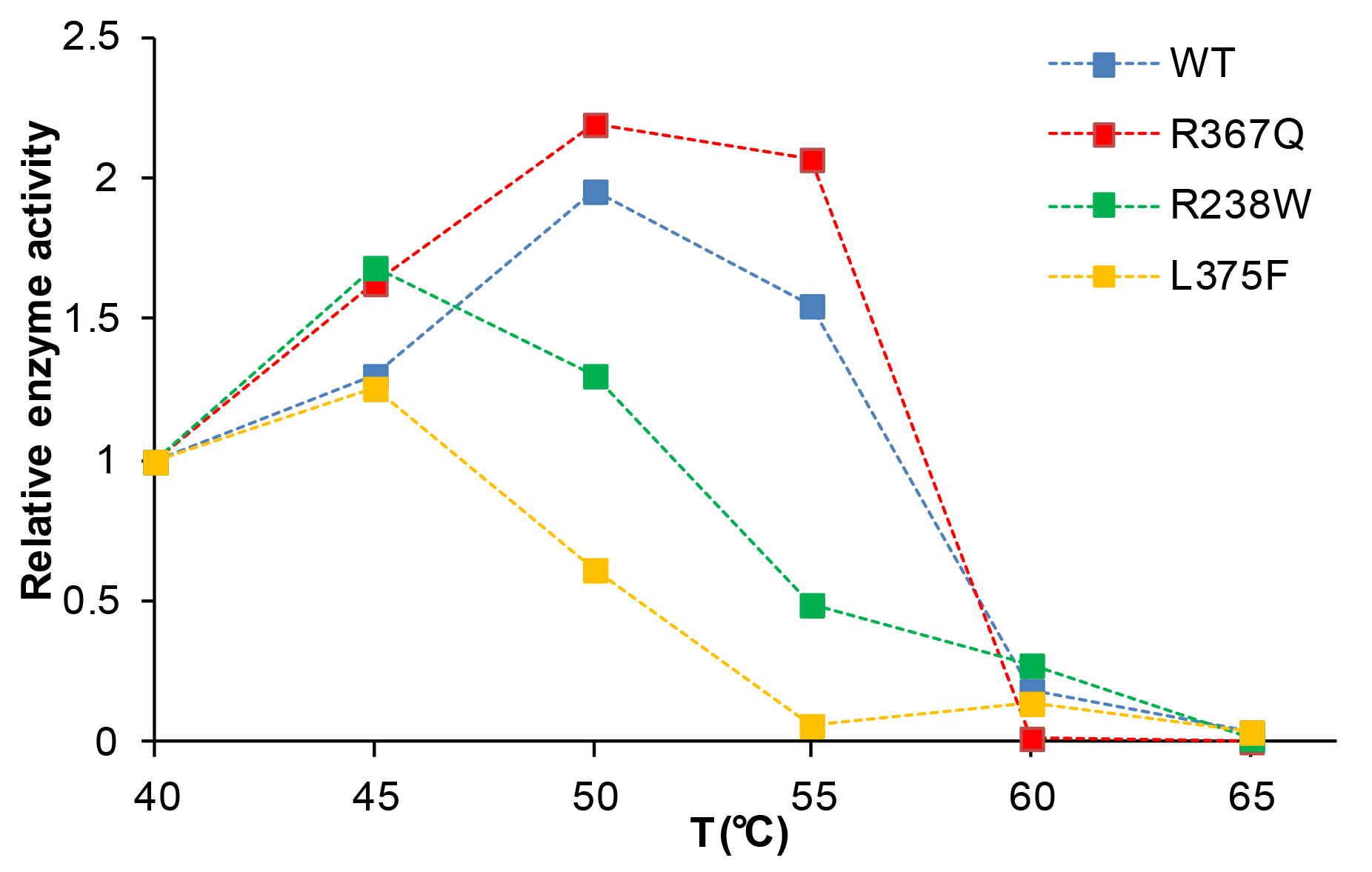

Supplement: Additional file 2: — Temperature dependence of cN-II catalytic activity. The activity values are relative to the values obtained at 40 °C with 1 mM IMP as substrate. The points represent mean values from two independent measurements. (TIF 452 kb) [file 12915_2016_313_MOESM2_ESM.tif]

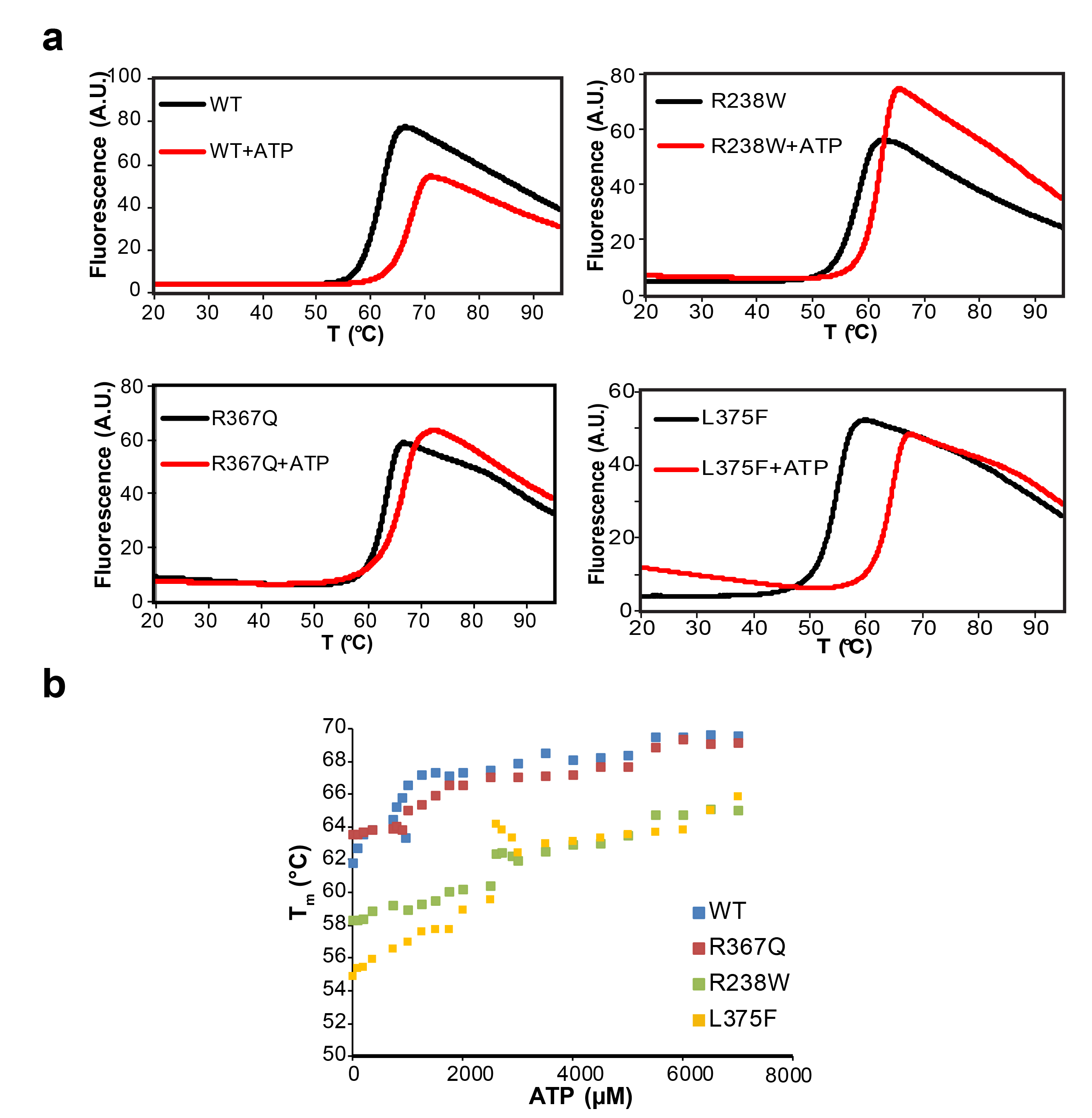

Supplement: Additional file 3: — Thermostability of cN-II proteins in the presence of ATP studied by differential scanning fluorimetry. a Representative curves of the wild-type and mutant proteins in the presence or absence of 3 mM ATP. b The T m values of the cN-II proteins under varying concentrations of ATP. Each point represents the mean value from two measurements. (TIF 1192 kb) [file 12915_2016_313_MOESM3_ESM.tif]

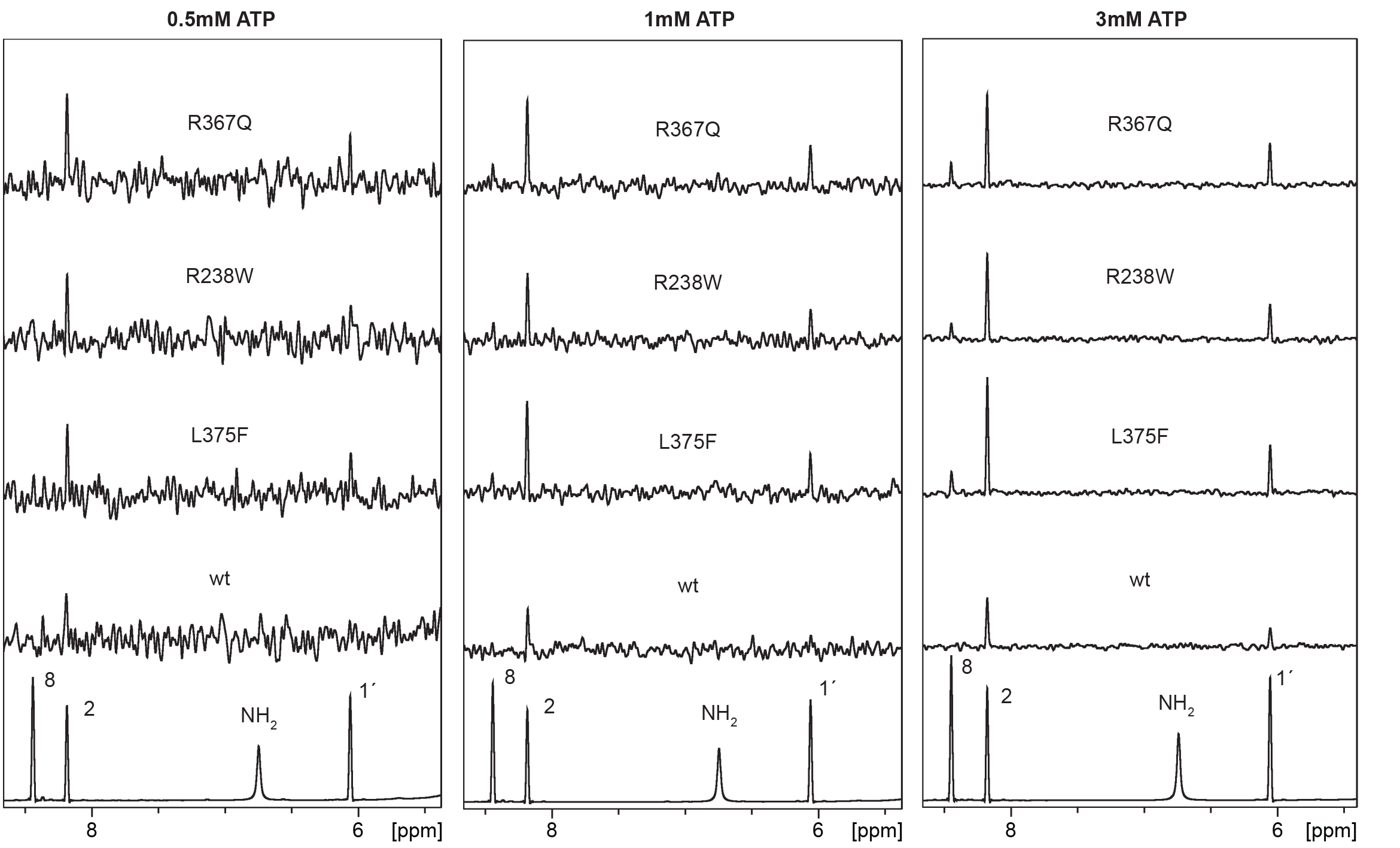

Supplement: Additional file 4: — 1H STD NMR spectra of ATP at various concentrations in the presence of cN-II variants. The ATP reference 1H NMR spectrum (standard atom numbering) is shown at the bottom. (TIF 1286 kb) [file 12915_2016_313_MOESM4_ESM.tif]

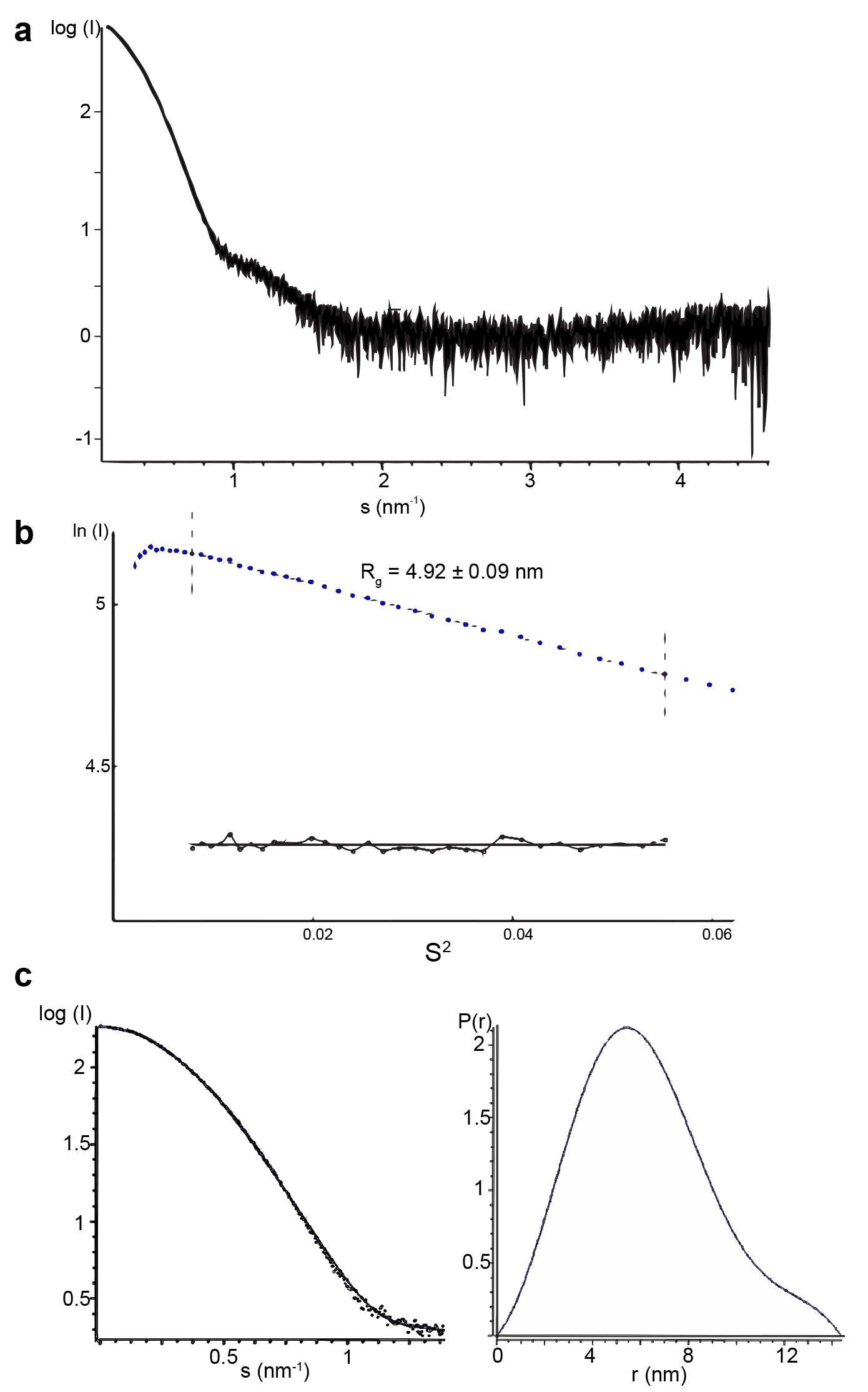

Supplement: Additional file 5: — Representative data from SAXS measurements: analysis of wild-type cN-II (1 mg/ml). a Processed scattering curve. b Guinier analysis. c Distance distribution analysis including calculation of the P (r) function. (TIF 836 kb) [file 12915_2016_313_MOESM5_ESM.tif]

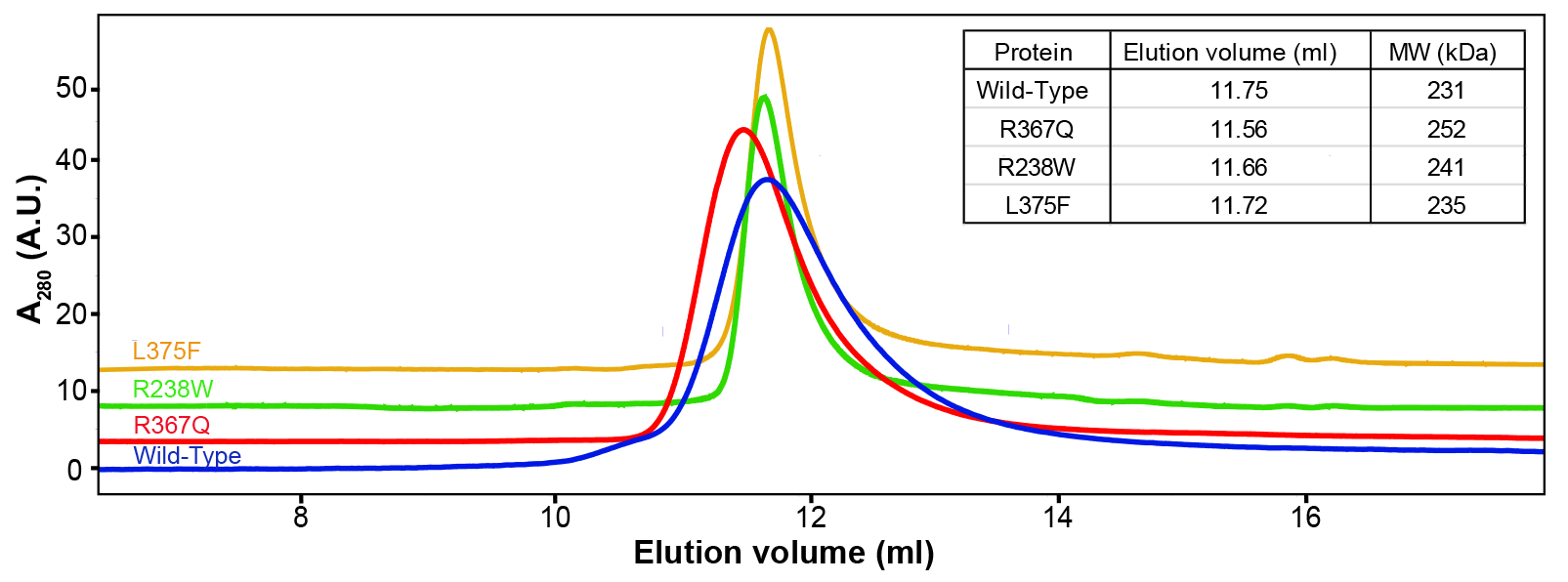

Supplement: Additional file 8: — Size exclusion chromatography of the C-terminally truncated cN-II proteins that were studied by X-ray crystallography. Each variant formed tetramers as reported for full-length proteins. The column was calibrated using a high molecular weight protein standard kit (Amersham Biosciences). (TIF 284 kb) [file 12915_2016_313_MOESM8_ESM.tif]

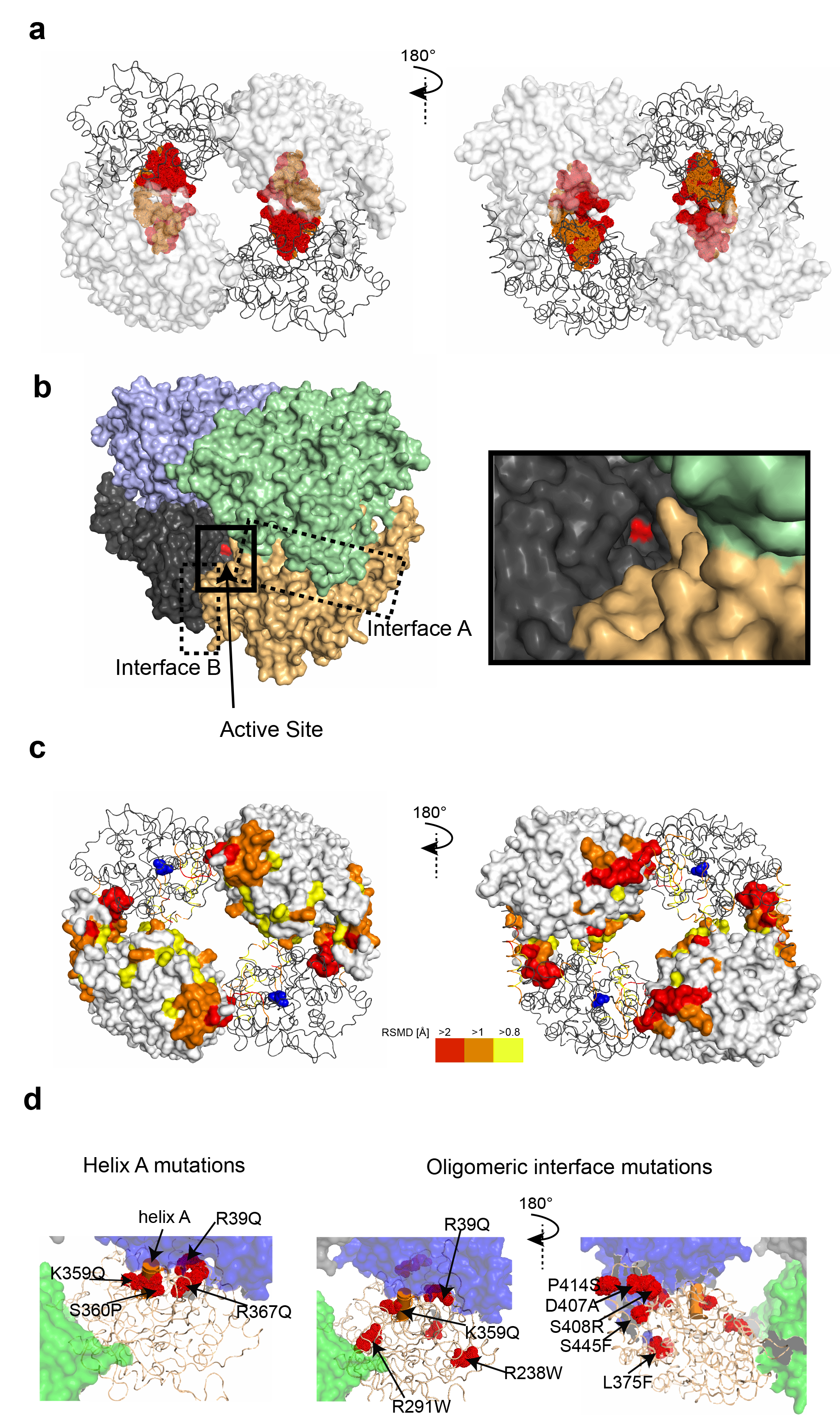

Supplement: Additional file 12: — Structural analysis demonstrates an important role for the oligomeric interface in cN-II allosteric regulation. a Position of helix A (red dots) and the ATP binding site (orange dots) within the cN-II tetramer. b The access channel towards the active site is formed by interface B. Each subunit is highlighted in a different color; the active site residue D52 is shown in red. c Superposition of wild type in its free state and in complex with ATP (PDB IDs 2XCX and 2XCW). Differences in positions of Cα atoms are highlighted in the structure of the apo form (PDB ID 2XCX). The active site residue (D52) is shown as blue spheres. d Location of ALL-specific mutations reveals two hotspot regions. Each subunit is depicted in a different color, and helix A is shown as an orange cylinder. Mutated residues are highlighted as red spheres within one subunit shown in ribbon representation (wheat colored). (TIF 10513 kb) [file 12915_2016_313_MOESM12_ESM.tif]

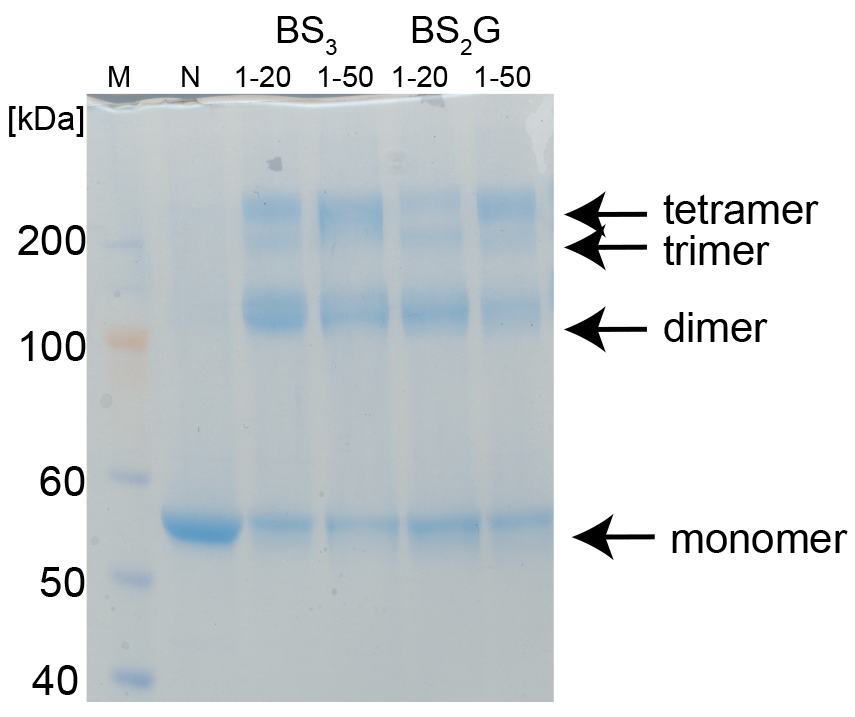

Supplement: Additional file 14: — Representative SDS-PAGE of the wild-type cN-II protein upon crosslinking reaction with bis(sulfosuccinimidyl)glutarate (BS2G) and bis(sulfo-succinimidyl)suberate (BS3). M and N refer to molecular weight marker and non-modified sample, respectively. The protein:crosslinker molar ratio is indicated at the top of each lane. The arrows indicate the number of subunits contributing to crosslinking products. (TIF 1253 kb) [file 12915_2016_313_MOESM14_ESM.tif]
