# Supplementary material for: Oligomeric interface modulation causes misregulation of purine 5´-nucleotidase in relapsed leukemia
Source: BMC Biol. 2016 Oct 19;14:91. doi: 10.1186/s12915-016-0313-y (PMC5070119; doi:10.1186/s12915-016-0313-y)
Supplement: Additional file 6: — Kinetic parameters of the C-terminally truncated enzymes in the presence and absence of ATP. The n value refers to the calculated Hill coefficient. (DOCX 15 kb) [file 12915_2016_313_MOESM6_ESM.docx]

| **Truncated** | **ATP** | **V_max_**  **[µmol.min^-1^mg^-1^]** | **K_m_ [mM]** | **n** | **kcat (s^-1^)** | **k_cat_/K_m_ [mM.s^-1^]** |
| --- | --- | --- | --- | --- | --- | --- |
| wild-type | - | 10.3 ± 2.2 | 32 ± 13 | 0.91 ± 0.13 | 11.5 ± 2.4 | 0.35 ± 0.16 |
| wild-type | + | 21.4 ± 1.9 | 5.4 ± 1.7 | 0.83 ± 0.11 | 23.8 ± 2.1 | 4.4 ± 1.4 |
| R367Q | - | 20.8 ± 2.2 | 5.6 ± 1.7 | 1.05 ± 0.26 | 23.3 ± 2.5 | 4.2 ±1.3 |
| R367Q | + | 24.9 ± 1.4 | 1.5 ± 0.4 | 0.84 ± 0.13 | 27.9 ± 1.5 | 18.8 ± 5.7 |
| R238W | - | 23.7 ± 2.4 | 11.1 ± 2.7 | 1.06 ± 0.17 | 26.5 ± 2.6 | 2.4 ± 0.6 |
| R238W | + | 19.5 ± 1.1 | 0.81 ± 0.22 | 0.85 ± 0.14 | 21.8 ± 1.2 | 25.7 ± 8.0 |
| L375F | - | 22.5 ± 1.0 | 3.1 ± 0.4 | 1.7 ± 0.3 | 25.1 ± 1.1 | 8.1 ± 1.4 |
| L375F | + | 22.1 ± 1.2 | 0.52 ± 0.16 | 0.80 ± 0.16 | 24.7 ± 1.4 | 48 ± 16 |

**Additional file 6.** **Kinetic parameters of C-terminally truncated enzymes in the presence and absence of ATP.** The 'n' value refers to the calculated Hill coefficient.
