# Supplementary material for: Oligomeric interface modulation causes misregulation of purine 5´-nucleotidase in relapsed leukemia
Source: BMC Biol. 2016 Oct 19;14:91. doi: 10.1186/s12915-016-0313-y (PMC5070119; doi:10.1186/s12915-016-0313-y)
Supplement: Additional file 7: — Differential scanning fluorimetry of the C-terminally truncated cN-II variants in the presence or absence of 3 mM ATP. Truncated proteins are stabilized upon ATP binding, as described for full-length variants. Mean values and standard deviations from duplicates of two independent measurements are listed. (DOCX 14 kb) [file 12915_2016_313_MOESM7_ESM.docx]

**Additional file 7**. **Differential scanning fluorimetry of the C-terminally truncated cN-II variants in the presence or absence of 3 mM ATP**. Truncated proteins are stabilized upon ATP binding, as described for full-length variants. Mean values and standard deviations from duplicates of two independent measurements are listed.

|  | T_m_ (°C) | |
| --- | --- | --- |
| 3 mM ATP | - | + |
| WT | 63.3 ± 0.5 | 69.0 ± 0.1 |
| R367Q | 64.2 ± 0.1 | 67.8 ± 0.1 |
| R238W | 60.7 ± 0.1 | 63.3 ± 0.4 |
| L375F | 57.4 ± 0.7 | 62.2 ± 0.3 |
