# Supplementary material for: Oligomeric interface modulation causes misregulation of purine 5´-nucleotidase in relapsed leukemia
Source: BMC Biol. 2016 Oct 19;14:91. doi: 10.1186/s12915-016-0313-y (PMC5070119; doi:10.1186/s12915-016-0313-y)
Supplement: Additional file 9: — Oligomeric interface of cN-II crystals analyzed by PISA. NHB and NSB represent the number of identified hydrogen bonds and salt bridges, respectively. ∆i G is the energy of solvation. (DOCX 15 kb) [file 12915_2016_313_MOESM9_ESM.docx]

**Additional file 9. Oligomeric interface of cN-II crystals analyzed by PISA.** N_HB_ and N_SB_ represent the number of identified hydrogen bonds and salt bridges, respectively. ∆^i^ G is the energy of solvation.

|  | **Protein** | **Interface area (Å^2^)** | **∆^i^ G (kcal/mol)** | **N_HB_** | **N_SB_** |
| --- | --- | --- | --- | --- | --- |
| Interface A | WT | 2376.3 | -21.5 | 16 | 10 |
|  | R367Q | 2053.8 | -10.3 | 32 | 14 |
|  | R238W | 2220.7 | -12.1 | 30 | 12 |
|  | L375F | 2132.8 | -9.8 | 30 | 16 |
| Interface B | WT | 1057.2 | -10 | 18 | 6 |
|  | R367Q | 900.2 | -10.4 | 14 | 0 |
|  | R238W | 864.9 | -10.1 | 10 | 0 |
|  | L375F | 922.3 | -10.2 | 10 | 0 |
