# Supplementary material for: Oligomeric interface modulation causes misregulation of purine 5´-nucleotidase in relapsed leukemia
Source: BMC Biol. 2016 Oct 19;14:91. doi: 10.1186/s12915-016-0313-y (PMC5070119; doi:10.1186/s12915-016-0313-y)
Supplement: Additional file 10: — Regions with perturbed deuteration kinetics analyzed in crystal structures using RINalyzer. The ‘+’ and ‘-’ symbols refer to the presence or absence of structural changes, respectively. (DOCX 15 kb) [file 12915_2016_313_MOESM10_ESM.docx]

**Additional file 10.**  **Regions with perturbed deuteration kinetics analyzed in crystal structures using RINalyzer.** The ‘+’ and ‘-’ symbols refer to the presence or absence of structural changes, respectively.

|  |  | **Conformational changes in the crystal structure analyzed by RINalyzer** | | |
| --- | --- | --- | --- | --- |
| **Segment** | **HDX kinetics in the mutants compared to the wild-type** | **R367Q** | **R238W** | **L375F** |
| 33-43 | faster | + | + | + |
| 47-54 | faster | + | - | + |
| 111-126 | faster | + | + | + |
| 182-191 | slower | + | + | + |
| 283-287 | slower | - | - | + |
| 293-302 | faster | + | + | + |
| 303-316 | slower | + | + | + |
| 370-381 | faster | - | - | + |
| 393-398 | faster | + | + | + |
| 419-440 | faster | + | + | + |
| 441-449 | faster | + | + | + |
| 454-460 | faster | + | + | + |
| 461-465 | faster | - | - | + |
| 469-476 | faster | + | + | + |
