# Supplementary material for: Oligomeric interface modulation causes misregulation of purine 5´-nucleotidase in relapsed leukemia
Source: BMC Biol. 2016 Oct 19;14:91. doi: 10.1186/s12915-016-0313-y (PMC5070119; doi:10.1186/s12915-016-0313-y)
Supplement: Additional file 11: — Cross-linking of the wild-type enzyme and R367Q mutant: modified residues identified and quantified using mass spectrometry. Relative abundance refers to a quantitative comparison between products obtained from wild-type and R367Q. The type of crosslinking (i.e., intermolecular, intramolecular or hanging) was inferred from the mass spectrometric data or from the crystal structure. Each number represents the mean value and standard deviation from three experiments. (DOCX 18 kb) [file 12915_2016_313_MOESM11_ESM.docx]

**Additional file 11**. **Cross-linking of the wild-type enzyme and R367Q mutant: modified residues identified and quantified using mass spectrometry****.** Relative abundance refers to a quantitative comparison between products obtained from wild-type and R367Q. The type of crosslinking (i.e., intermolecular, intramolecular or hanging) was inferred from the mass spectrometric data or from the crystal structure. Each number represents the mean value and standard deviation from three experiments.

|  |  |  | **Relative abundance (wild-type compared to R367Q)** | | | |
| --- | --- | --- | --- | --- | --- | --- |
|  |  |  | **Protein : BS2G molar ratio** | | **Protein : BS3 molar ratio** | |
| **Peptide** | **Modified residues** | **Type of crosslinking** | **1:20** | **1:50** | **1:20** | **1:50** |
| 343-361 | K344; K359 | Intra. | 77-23 ± 20 | 88-12 ± 18 | 47-53 ± 24 | 65-35 ± 1 |
| 345-362 | K359; K361 | Intra. | 37-63 ± 4 | 48-52 ± 13 | 20-80 ± 11 | 19-81 ± 5 |
| 345-361 | K359 | Hanging | 24-76 ± 11 | 34-66 ± 15 | 17-83 ± 12 | 14-86 ± 18 |
| 343-359 | K344 | Hanging | 80-20 ± 16 | 82-18 ± 10 | 94-6 ± 4 | 76-24 ± 13 |
| 135-144; 423-425 | K140; K424 | Intra. | 42-58 ±4 | 47-53 ±18 | 38-62 ± 7 | 32-68 ±14 |
| 40-47; 423-425 | K45; K424 | Inter. | 33-67 ±4 | 31-69 ±17 | 32-68 ±11 | 33-67 ±4 |
| 245-258; 309-313 | K254; K311 | Intra. | 38-62 ±4 | 45-55 ±17 | 39-61 ± 9 | 39-61 ± 23 |
| 245-258; 304-311 | K254; K308 | Intra. | 49-51 ± 5 | 56-44 ± 8 | 32-68 ± 11 | 32-68 ± 9 |
| 40-47; 343-359 | K45; K344 | Intra. | 62-38 ± 6 | 68-32 ± 18 | 52-48 ± 5 | 43-57 ± 1 |
| 510-521 | K515; K520 | Intra. | 48-52 ± 5 | 55-45 ± 11 | 56-44 ± 3 | 59-41 ± 12 |
| 26-28; 229-238 | K26; K231 | Intra. | 43-57 ± 4 | 42-58 ± 17 | 48-52 ± 17 | 41-59 ± 19 |
| 304-313 | K308; K311 | Intra. | n.d. | n.d. | 46-54 ± 4 | 42-58 ± 14 |
| 40-47; 135-144 | K45; K140 | Inter. | n.d. | n.d. | 40-60 ± 1 | 32-68 ± 16 |
| 203-215; 309-313 | K211; K311 | Inter./Intra. | n.d. | n.d. | 39-61 ± 7 | 35-65 ± 15 |
| 203-215; 245-258 | K211; K254 | Inter./Intra. | n.d. | n.d. | 41-59 ± 4 | 38-62 ± 10 |
| 40-47 | K45 | Hanging | 51-49 ± 5 | 61-39 ± 6 | 47-53 ± 5 | 63-37 ± 30 |
| 135-144 | K140 | Hanging | 49-51 ± 2 | 45-55 ± 25 | 45-55 ± 4 | 50-50 ± 14 |
| 229-235 | K231 | Hanging | n.d. | 43-57 ± 6 | 51-49 ± 6 | 53-47 ± 20 |
| 510-520 | K515 | Hanging | 53-47 ± 5 | 44-56 ± 12 | 56-44 ± 14 | 72-28 ± 4 |
| 241-254 | K244 | Hanging | 46-54 ± 5 | 37-63 ± 11 | 40-60 ± 4 | 37-63 ± 17 |
| 218-228 | K224 | Hanging | n.d. | n.d. | 48-62 ± 8 | 38-62 ± 4 |
| 178-195 | K186 | Hanging | n.d. | n.d. | 44-56 ± 6 | 48-52 ± 13 |

n.d. not detected
