# Supplementary material for: Oligomeric interface modulation causes misregulation of purine 5´-nucleotidase in relapsed leukemia
Source: BMC Biol. 2016 Oct 19;14:91. doi: 10.1186/s12915-016-0313-y (PMC5070119; doi:10.1186/s12915-016-0313-y)
Supplement: Additional file 13: — Crystal parameters, data collection and refinement statistics. (DOCX 20 kb) [file 12915_2016_313_MOESM13_ESM.docx]

**Additional file 13.** **Crystal parameters, data collection and refinement statistics**

| **Data collection statistics** | | R367Q | R238W | L375F |
| --- | --- | --- | --- | --- |
| Space group | | *I*222 | *I*222 | *I*222 |
| Cell parameters (Å, º) | | a = 91.5 b = 126.5 c = 130.3 | a = 91.3 b = 126.5 c = 130.5 | a = 91.4 b = 126.0 c= 130.8 |
|  |  | α = β = γ = 90 | α = β = γ = 90 | α = β = γ = 90 |
| Number of molecules in AU | | 1 | 1 | 1 |
| Wavelength (Å) | | 0.918409 | 0.918409 | 0.918409 |
| Resolution (Å)^a^ | | 48.948-1.738 | 48.305- 1.839 | 48.989-1.643 |
|  |  | (1.747-1.738) | (1.849-1.839) | (1.651-1.643) |
| Number of unique reflections^a^ | | 76,477 (11,173) | 65,417 (10,467) | 90,521 (13,998) |
| Multiplicity^a^ | | 5.2 (3.7) | 3.7 (3.6) | 4.4 (4.2) |
| Completeness (%)^a^ | | 98.2 (89.6) | 99.4 (99.1) | 98.6 (95.1) |
| R_meas_ (%)^a,b^ | | 5.3 (70.7) | 5.5 (71.0) | 6.4 (66.9) |
| Average *I*/σ (*I*)^a^ | | 20.1 (1.9) | 18.5 (2.0) | 14.3 (1.9) |
| Wilson B (Å^2^) | | 36.3 | 35.6 | 30.3 |
| **Refinement statistics** |  |  |  |  |
| Resolution range (Å)^a^ | | 48.33-1.79 | 45.42-1.84 | 48.99-1.64 |
|  |  | (1.835-1.788) | (1.887-1.839) | (1.685-1.643) |
| No. of reflections in working set^a^ | | 67,541 (4,594) | 63,308 (4,593) | 88,417 (5,868) |
| No. of reflections in test set^a^ | | 3,555 (241) | 2,101 (153) | 2,100 (139) |
| R value (%)^a,c^ | | 17.1 (34.6) | 17.7 (34.1) | 17.5 (36.2) |
| R_free_ value (%)^a,d^ | | 19.2 (35.4) | 20.5 (38.2) | 19.6 (35.2) |
| RMSD bond length (Å) | | 0.012 | 0.013 | 0.013 |
| RMSD angle (º) | | 1.47 | 1.56 | 1.60 |
| Number of atoms in AU | | 4,406 | 4,225 | 4,323 |
| Number of protein atoms in AU | | 4,020 | 3,936 | 3,989 |
| Number of water molecules in AU | | 340 | 209 | 254 |
| Mean B value (Å^2^)^e^ | | 33.1 | 38.4 | 29.7 |
| **Ramachandran plot statistics:** | |  |  |  |
| Residues in favored regions (%)^f^ | | 98.2 | 97.3 | 98.2 |
| Residues in allowed regions (%)^f^ | | 1.2 | 2.5 | 1.6 |
| Ramachandran outliers (%)^f^ | | 0.6 | 0.2 | 0.2 |

^a^ values in parentheses refer to the highest resolution shell

^b^ R_meas_ = Σ_hkl_{N(hkl)/[N(hkl) -1]}^1/2^ Σ_i_ |I_i_ (hkl)-〈 I(hkl)〉 |/Σ_hkl_ Σ_i_ I_i_ (hkl), where 〈I(hkl)〉 is the mean of the N(hkl) individual measurements I_i_ (hkl) of the intensity of reflection hkl

^c^ R = ∑ |F_o_ - F_c_| / ∑ |F_o_|, where F_o_ and F_c_ are the observed and calculated structure factors, respectively.

^d^ R_free_ is equivalent to R but is calculated for 5% of the reflections chosen at random and omitted from the refinement process [ Brunger, A.T. Free R value: a novel statistical quantity for assessing the accuracy of crystal structures. *Nature* 355, 472-475 (1992)]

^e^ as determined by Baverage [Winn, M.D. et al. Overview of the CCP4 suite and current developments. *Acta Crystallographica Section D-Biological Crystallography* 67, 235-242 (2011)]

^f^ as determined by MolProbity [Lovell, S.C. et al. Structure validation by C alpha geometry: phi,psi and C beta deviation. *Proteins-Structure Function and Genetics* 50, 437-450 (2003)]
